# Modulation of TCR Signaling by Tyrosine Phosphatases: From Autoimmunity to Immunotherapy

**DOI:** 10.3389/fcell.2020.608747

**Published:** 2020-12-09

**Authors:** Patricia Castro-Sanchez, Alexandra R. Teagle, Sonja Prade, Rose Zamoyska

**Affiliations:** Ashworth Laboratories, Institute of Immunology and Infection Research, University of Edinburgh, Edinburgh, United Kingdom

**Keywords:** phosphatase, T cell, TCR signaling, autoimmunity, immunotherapy

## Abstract

Early TCR signaling is dependent on rapid phosphorylation and dephosphorylation of multiple signaling and adaptor proteins, leading to T cell activation. This process is tightly regulated by an intricate web of interactions between kinases and phosphatases. A number of tyrosine phosphatases have been shown to modulate T cell responses and thus alter T cell fate by negatively regulating early TCR signaling. Mutations in some of these enzymes are associated with enhanced predisposition to autoimmunity in humans, and mouse models deficient in orthologous genes often show T cell hyper-activation. Therefore, phosphatases are emerging as potential targets in situations where it is desirable to enhance T cell responses, such as immune responses to tumors. In this review, we summarize the current knowledge about tyrosine phosphatases that regulate early TCR signaling and discuss their involvement in autoimmunity and their potential as targets for tumor immunotherapy.

## Introduction

Effective T cell responses require naïve T cell activation, proliferation and differentiation into effector and memory cells. Naïve T cells are activated when their T cell receptors (TCR) interact with a specific antigen presented by the major histocompatibility complex (MHC) on an antigen presenting cell (APC). In this process the extracellular engagement sensed by the TCR must be transmitted to the inside of the cell, whereupon signaling must propagate rapidly and alter gene expression to induce a lasting cellular response. After the response has been triggered, signaling must be turned off. Therefore, TCR signal propagation must be fast and reversible. These qualities are provided by post-translational protein modifications (reviewed in Deribe et al., 2010) that alter the properties of a protein by reversible addition of a chemical group such as a phosphate (phosphorylation) or another protein such as ubiquitin (ubiquitination) to one or more amino acids. Tyrosine phosphorylation is one of the main, although by no means only, post-translational modification driving early TCR signaling.

## Tyr Phosphatases

Tyrosine phosphorylation controls a wide range of cellular processes in eukaryotic cells and is regulated by the opposing dynamic activities of tyrosine kinases and phosphatases. In fact, there is a similar number of both groups of enzymes in the human genome: 84 genes encode for catalytically active tyrosine kinases (Robinson et al., 2000; Manning et al., 2002) and 74 for phosphatases known to dephosphorylate Tyrosine residues (hereafter Tyr phosphatases), all of which have mouse orthologs (Alonso and Pulido, 2016). This review will focus on Tyr phosphatases, which belong to the protein tyrosine phosphatase (PTP) superfamily, also known as the PTPome (Alonso et al., 2004; Alonso and Pulido, 2016).

Tyr phosphatases share a catalytic mechanism, in which the catalytic residue performs a nucleophilic attack on the phosphate group of the substrate, leading to the formation of an intermediate that is subsequently hydrolyzed (Tonks, 2006). The catalytic residue is generally cysteine, with a few exceptions such as the STS phosphatases, in which aspartic acid performs the nucleophilic attack (Alonso and Pulido, 2016). Tyr phosphatases are very diverse in terms of structural domains and motifs, which contributes to their heterogeneous subcellular localization. A subgroup of receptor Tyr phosphatases have a transmembrane domain that places them on the plasma membrane, where they can control cellular responses to extracellular stimuli. Some cytoplasmic phosphatases have an SH2 domain that allows them to bind Tyr-phosphorylated proteins, which are often transmembrane receptors and adaptors. This provides a rapid and reversible mechanism to direct phosphatases to the inner face of the plasma membrane, where they can regulate membrane proximal signaling in a dynamic manner. Phosphatases with a FERM domain interact with actin and localize at the interface between the plasma membrane and the cortical cytoskeleton. Phosphatases with a nuclear localization and/or a nuclear export signal are restricted to the nucleus or to the cytoplasm, or shuttle between both compartments. This diversity is relevant since it gives the phosphatase family the potential to regulate any cellular process in any subcellular region.

### Tyr Phosphatases in T Cells

The essential role of tyrosine phosphatases in regulation of T cell activation was highlighted by early experiments in which pervanadate, a potent inhibitor of tyrosine phosphatases, was administered to T cells *in vitro* (Heffetz et al., 1990). Treatment of T cells with pervanadate resulted in rapid activation of the cells, including induction of proximal TCR signaling and production of IL-2, despite the absence of TCR engagement (Secrist et al., 1993). This finding shows that, taken as a whole, phosphatases dominate over kinases to maintain T cells in a resting state in the absence of antigenic stimulation. However, the picture is much more nuanced, as multiple phosphatases are involved, with potentially overlapping roles, to regulate both T cell homeostasis and responses. In addition, some phosphatases are required to initiate TCR signaling, such as CD45, while others amplify it, such as low molecular weight phosphotyrosine protein phosphatase, LMPTP. Clinical observations also point to an important role of Tyr phosphatases in T cell signaling and immunity. It has been demonstrated that perturbations in the expression or function of some Tyr phosphatases can lead to immunodeficiency on the one hand, when the altered phosphatase, for example CD45, is required for TCR signaling (Kung et al., 2000; Tchilian et al., 2001), or on the other hand, autoimmunity, when the altered phosphatase is a negative regulator of TCR signaling, for example, protein tyrosine phosphatase (PTP)N22 (Todd et al., 2007; Bottini and Peterson, 2014). These observations underscore how phosphatases are key in maintaining a delicate balance between immune responses that provide protection from infectious agents, while maintaining self-tolerance that prevents autoimmune disorders.

**FIGURE 1:**
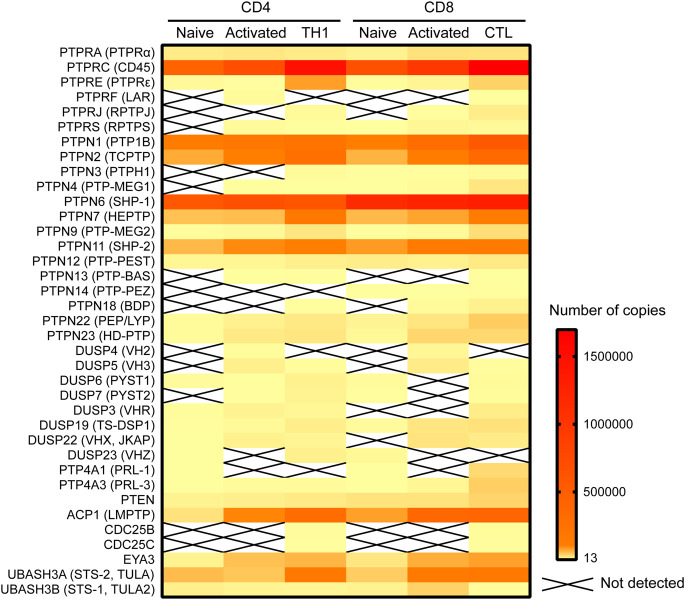
Expression of Tyr phosphatases in primary murine CD4 and CD8 cells. Data on the number of copies of Tyr phosphatases was extracted from the proteomic study by Howden et al. (2019) and visualized in a heatmap using Prism software.

Of the 74 Tyr phosphatases in the genome, 37 were detected in a recent proteomic study of murine mature CD4 and CD8 T cells (Howden et al., 2019; Figure 1). Of note, this study found that the abundance of several phosphatases was modulated during differentiation of murine CD8 and CD4 T cells and/or T cell activation. Such regulated expression is consistent with previous data on human CD4 T cells (Castro-Sanchez et al., 2017), and highlights that both the number of phosphatases and the protein abundance of each expressed phosphatase shapes the T cell phenotype and the manner in which a T cell responds to antigen. Alteration of protein abundance, however, takes at least minutes if not hours or days to achieve, while early TCR signaling occurs within seconds. In this temporal scale, spatial regulation of proteins is the most efficient mechanism to control local protein concentrations. Early TCR signaling takes place in the context of the immunological synapse, a highly organized, dynamic contact between a T cell and an APC (reviewed in Dustin, 2014). To regulate TCR proximal signaling events, phosphatases must polarize to the area of the interaction, and position in close proximity to their substrates. The substrates are often transmembrane proteins, such as the ζ-chain, or cytoplasmic proteins localized at the inner face of the plasma membrane, such as the SRC-family kinase LCK. How do cytoplasmic phosphatases reach these substrates? Which adaptors or scaffolding proteins aid in the localization of phosphatases that themselves may lack specific localization domains or motifs? These questions have been frequently overlooked but answering them would greatly improve our understanding of the often nuanced manner by which Tyr phosphatases regulate T cell activation in health and disease.

To date, 15 Tyr phosphatases have been reported to regulate molecules involved in early TCR signaling (Table 1). In this review we will discuss their role in controlling proximal TCR signaling, their implication in autoimmunity and their potential as targets in immunotherapy.

**TABLE 1:** Tyr phosphatases reported to regulate early TCR signaling.

**Gene**	**Protein**	**Substrate in early TCR signaling**	**Localization features or domains**
PTPRA	RPTPα	LCK, FYN	TM Receptor phosphatase
PTPRC	CD45	LCK, FYN, ζ-chain	TM Receptor phosphatase
PTPRE	RPTPε	LCK	TM Receptor phosphatase
PTPRH	SAP-1	LCK	TM Receptor phosphatase
PTPRJ	CD148	LCK	TM Receptor phosphatase
PTPN2	TCPTP	LCK, FYN	Nuclear and ER localization signals
PTPN3	PTPH1	ζ-chain	FERM domain
PTPN4	PTP-MEG1	ζ-chain	FERM domain
PTPN6	SHP1	LCK, ζ-chain, ZAP70	SH2 domains
PTPN11	SHP2	ZAP70, CD28,	SH2 domains
PTPN22	LYP	LCK, ζ-chain, ZAP70	Polyproline regions
DUSP22	VHX	LCK	Myristoylation signal
ACP1	LMPTP	ZAP70	None defined
UBASH3A	STS-2, TULA	ZAP70	UBA, SH3
UBASH3B	STS-1, TULA2	ZAP70	UBA, SH3

*TM, Transmembrane; ER, Endoplasmic reticulum; FERM, protein 4.1, ezrin, radixin, moesin; SH2, Src Homology 2; UBA, Ubiquitin-Associated; SH3, Src Homology 3.*

### Regulation of Early Tcr Signaling by Tyr Phosphatases

Signaling downstream of the TCR occurs through a network of rapid phosphorylation events on tyrosine residues of several effector and adaptor proteins (reviewed in Courtney et al., 2018). The TCR lacks intrinsic enzymatic activity, hence it relies on the SRC-family kinases LCK and FYN to initiate signaling. LCK phosphorylates CD3 and ζ-chains on their immunoreceptor tyrosine-based activation motifs (ITAMs) (Straus and Weiss, 1992; van Oers et al., 1996). These serve as docking sites for the recruitment of the 70 KDa ζ-chain associated protein kinase, ZAP70, to the TCR, where it is phosphorylated and activated by LCK (Chan et al., 1992; van Oers et al., 1996). Active ZAP70 phosphorylates, amongst other substrates, the scaffold protein linker for activation of T cells (LAT), which leads to the formation of a molecular complex that induces further distal signaling, resulting in T cell activation and effector function (Sommers et al., 2004). By regulating these proximal TCR signaling events, Tyr phosphatases determine activation thresholds and signal intensity and duration.

### Regulation of SRC Family Kinases to Set the Activation Threshold and Maintain Peripheral Tolerance

Survival and functionality of naïve T cells in the periphery requires continuous tonic signals from self-peptide loaded MHC molecules (van Oers et al., 1993; Stefanova et al., 2002). However, this tonic signaling must not trigger cell activation, otherwise autoimmune pathology may develop. A precise threshold of T cell activation must therefore be set to ensure that naïve T cells are not activated by self-antigens but are able to respond to foreign antigens. Precisely how this equilibrium is maintained by subtle interactions between multiple signaling molecules is incompletely understood. A key initial trigger that has been well described is the regulation of the activity of the SRC-family kinases LCK and FYN (Seddon and Zamoyska, 2002) by phosphor/dephosphorylation of key residues (Box 1).

**Box 1. Regulatory mechanism of key kinases involved in early TCR signaling transduction.**

**Src family kinases LCK and FYN.** LCK activity is regulated by phosphorylation of two key residues, Y505 and Y394 (Yamaguchi and Hendrickson, 1996; Boggon and Eck, 2004). Phosphorylation of Y505 in the LCK C-terminal domain by the kinase CSK prompts an inhibited, *closed* conformation. Dephosphorylation of this inhibitory residue raises a *primed* conformation (Bergman et al., 1992), which allows trans-autophosphorylation on Y394 in the activation loop, leading to the fully active *open* conformation. A fourth conformation with both Y394 and Y505 phosphorylated has been found in T cells, and *in vitro* data suggests that this conformation is also active (Nika et al., 2010). FYN is regulated in a very similar way as LCK (Salmond et al., 2009). Phosphorylation of the inhibitory Y528 by CSK inactivates it, while dephosphorylation of this residue allows autophosphorylation on the activating residue Y417, resulting in full activation. Upon TCR stimulation, active LCK can also phosphorylate FYN^Y417^, activating it (Filipp et al., 2008).

**ZAP70**. The activation of ZAP70 is regulated by localization and phosphorylation (reviewed in Au-Yeung et al., 2018; Figure 4). Binding of the SH2 domains of ZAP70 to pTyr in ITAMs of the ζ chains induces a conformational change in ZAP70 that aligns the SH2 domains, leading to increased affinity for the phosphorylated ITAMs, and exposes its activation loop, while also localizing ZAP70 in the proximity of LCK. Then, LCK phosphorylates Y315 and Y319 on the activation loop, and phosphorylation of Y493 on the kinase domain either by LCK or by autophosphorylation leads to full activation of ZAP70. Phosphorylation of Y292 on the activation loop and of Y492 on the kinase domain of ZAP70 dampen kinase activity, although the mechanism is not fully understood.

#### CD45

**FIGURE 2:**
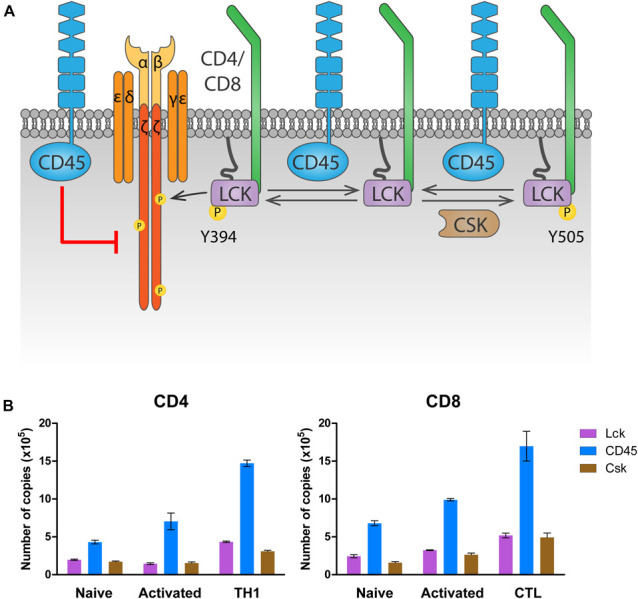
Regulation of LCK by CD45 and CSK. **(A)** Regulation of tonic signaling. In absence of antigen stimulation, basal LCK activity ensures tonic ζ chain phosphorylation and signaling needed for naïve T cell survival. Phosphorylation of LCK on Y505 by CSK prompts an inhibited conformation (right). CD45 dephosphorylates Y505 to raise a *primed* conformation (center), which allows trans-autophosphorylation on Y394, leading to the fully active *open* conformation that phosphorylates the ζ chain (left). To avoid excessive LCK activation, CD45 dephosphorylates LCK on Y394. At the same time, CD45 dephosphorylates the ζ chain, inhibiting downstream signaling in the absence of antigen stimulation. **(B)** Number of LCK, CD45 and CSK molecules in CD4 and CD8 T cells. Data was extracted from the proteomic study by Howden et al. (2019) and visualized using Prism software.

The highly expressed receptor-type tyrosine-protein phosphatase C (commonly known as CD45, encoded by the gene *PTPRC*) keeps LCK in a poised activation state in naïve T cells by dephosphorylating LCK^Y505^ (Stone et al., 1997; Seavitt et al., 1999; Figure 2A). This maintains basal levels of ζ-chain phosphorylation and provides tonic signaling needed for survival of naïve T cells (reviewed in Zamoyska et al., 2003). At the same time, and to prevent naïve T cell activation in the absence of antigen stimulation, CD45 also dephosphorylates LCK^pY394^, inactivating it (D’Oro et al., 1996). The latter dephosphorylation, however, requires the high CD45 expression levels displayed by mature T cells. In fact, experiments manipulating CD45 expression have shown that T cells with very low amounts of CD45 had impaired T cell responses, because LCK is not sufficiently activated by dephosphorylation of pY505 (McNeill et al., 2007; Zikherman et al., 2010). Intermediate amounts of CD45 cause T cell hyperactivation, since CD45 abundance is enough to activate LCK through pY505 dephosphorylation, but not to limit its activation through pY394 dephosphorylation. Only the high physiologic CD45 abundance ensures sufficient primed LCK protein to trigger a T cell response while preventing T cell hyperactivation in the absence of antigen. This model provides a rationale for the consistent relative protein copy number found in several different primary T cell subsets, between LCK, CD45 and C-terminal Src kinase (CSK), the kinase responsible for Lck^Y505^ phosphorylation (Figure 2B). In both CD4 and CD8 T cells, a ratio of at least two CD45 molecules are found per LCK molecule to control LCK activity. In contrast, one molecule of CSK per LCK molecule seems to be sufficient to regulate LCK^Y505^ phosphorylation. Once antigen is encountered, there is evidence that segregation of CD45 from ligated TCRs in the immunological synapse is required to allow persistent phosphorylation of the ζ-chain that triggers TCR signaling (Leupin et al., 2000; Davis and van der Merwe, 2006; Varma et al., 2006; Cordoba et al., 2013; Chang et al., 2016). In fact, CD45-mediated tonic dephosphorylation of the ζ-chains in resting T cells helps prevent activation in the absence of antigen (Figure 2A; Courtney et al., 2019).

#### RPTPε

CD45 is one transmembrane receptor Tyr phosphatase with a well characterized role in regulation of SRC-family kinases in mature T cells. Further investigation into the function of other receptor Tyr phosphatases is likely to reveal new players in this regulation. Some experimental evidence has been reported for receptor-type tyrosine-protein phosphatase ε (RPTPε), receptor-type tyrosine-protein phosphatase H (RPTPH) and receptor-type tyrosine-protein phosphatase J (RPTPJ) so far. RPTPε (encoded by the gene *PTPRE*) has been proposed as a positive regulator of LCK activity, based on the observation that cells with low levels of RPTPε (induced by incubation of T cells with a hepatitis C virus-derived small RNA) showed reduced phosphorylation on LCK^Y394^ and downstream molecules upon TCR stimulation, which resulted in impaired T cell activation (Bhattarai et al., 2015; Bhattarai et al., 2017). Analysis of the phenotype of *PTPRE* knockout cells would provide important further validation of its role in the regulation of LCK activity.

#### RPTPH

RPTPH (also known as SAP-1, encoded by the gene *PTPRH*) interacts with LCK both *in vitro* and *in vivo*, and overexpression of this phosphatase resulted in decreased phosphorylation of LCK^pY394^ and impaired T cell activation (Ito et al., 2003), suggesting that LCK is a direct substrate. RPTPH, however, was not detected in primary murine T cells (Howden et al., 2019) or Jurkat cells (Ito et al., 2003), hence a physiological role for this phosphatase in T cell regulation is unlikely.

#### RPTPJ

RPTPJ (also known as CD148, encoded by the gene *PTPRJ*), when overexpressed in Jurkat cells, bound to LCK and dephosphorylated both pY394 and pY505 residues, which resulted in a net inhibitory effect on LCK activity (Stepanek et al., 2011). Of note, RPTPJ was not detected in murine naïve T cells, while human naïve T cells express a significant amount (Stepanek et al., 2011; Castro-Sanchez et al., 2017). RPTPJ expression is induced and upregulated in murine and human effector T cells, respectively (Figure 1; Castro-Sanchez et al., 2017; Howden et al., 2019), so it may play a role in the regulation of T cell effector responses rather than in naïve T cell activation. RPTPJ knockout mice had no obvious phenotype with regard to T cell development, but T cell activation and recall responses in lineage specific knockouts have not yet been addressed for RPTPJ (Zhu et al., 2008).

#### SHP-1

Four cytoplasmic Tyr phosphatases are known to contribute to antigen discrimination and tolerance through dephosphorylation of Src family kinases on their activatory residues: Src homology 2-containing phosphatase 1 (SHP-1) (Stefanova et al., 2003), dual specificity protein phosphatase 22 (DUSP22) (Li et al., 2014), protein tyrosine phosphatase non-receptor type 2 (PTPN2) (Wiede et al., 2011) and protein tyrosine phosphatase non-receptor type 22 (PTPN22) (Cloutier and Veillette, 1999; Gjorloff-Wingren et al., 1999; Wu et al., 2006). Of them, only DUSP22 (also known as JKAP or VHX) is permanently located at the inner face of the plasma membrane, due to co-translational and irreversible myristoylation on its N-terminal Glycine (Schwertassek et al., 2010). In contrast, SHP-1, PTPN2 and PTPN22 must be recruited to the immunological synapse in an inducible way, which allows for spatial regulation of their activities.

SHP-1 (encoded by the gene *PTPN6*) is recruited to the immunological synapse by its SH2 domains, which only bind Tyr phosphorylated proteins, such as the chains of the TCR complex. In fact, SHP-1 was reported to be recruited to the TCR upon stimulation with a TCR antagonist (Stefanova et al., 2003), where it dephosphorylated LCK on pY394 to inhibit the response to the antagonist. In contrast, binding of an agonist rapidly activated ERK, which blocked interaction of SHP-1 with LCK by phosphorylating LCK on serine 59, allowing downstream signaling. These data suggest that SHP-1 may be important for T cells to discriminate between TCR agonists and antagonists. However, T cell specific deletion of SHP-1 resulted in a mild phenotype in terms of T cell activation and showed that SHP-1 is also involved in T cell differentiation and AKT signaling (Fowler et al., 2010; Johnson et al., 2013; Mercadante and Lorenz, 2017). Studies using knockdown strategies have also shown that SHP-1 induces T cell adhesion and mediates IL-10 signaling in T cells (Taylor et al., 2007; Azoulay-Alfaguter et al., 2017). The involvement of SHP-1 in so many diverse functions provides a rationale for its high expression in primary T cells. In fact, SHP-1 is the most abundant Tyr phosphatase in naïve T cells and is only outpaced by CD45 increased expression following T cell stimulation (Figure 1). Its putative involvement in diverse signaling pathways might be an issue when considering SHP-1 as a target in immunotherapy (see section “Concluding Remarks”).

#### PTPN2

The spatial regulation of PTPN2 and PTPN22 remains poorly understood, despite their physiologic relevance. PTPN2 (also known as TCPTP) is important for establishing an appropriate T cell activation threshold that ensures tolerance (Wiede et al., 2011). It was suggested that PTPN2 regulates TCR signaling by dephosphorylation of SFKs, since a PTPN2 substrate-trapping mutant overexpressed in COS-1 cells bound LCK and FYN (Wiede et al., 2011). Whether this interaction takes place in a physiologic setting and how PTPN2 would reach these substrates in T cells remain unclear. The two described PTPN2 splicing variants, p45 and p48, localize to the nucleus (due to the presence of a nuclear localization signal) and to the endoplasmic reticulum (which requires the 19 C-terminal residues of the protein), respectively (Lorenzen et al., 1995). Small amounts of PTPN2 might reach the inner face of the plasma membrane and be stabilized there by its basic C-terminal residues, and additional mechanisms might translocate it to the immune synapse in an inducible manner. The use of fractionation techniques and microscopy would help clarify PTPN2 localization and how it regulates T cell activation thresholds.

#### PTPN22

**FIGURE 3:**
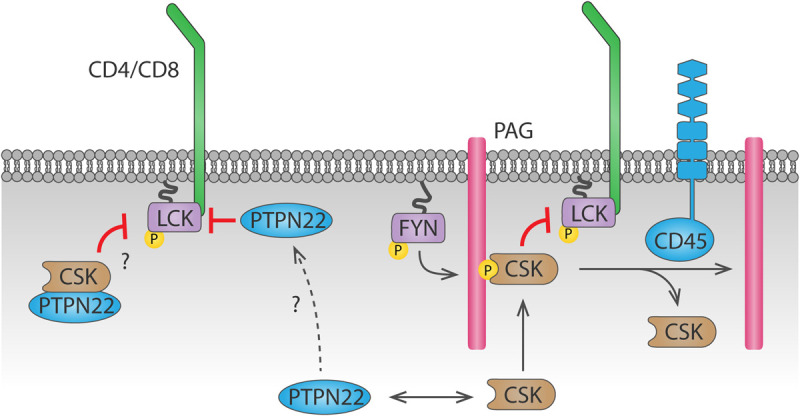
Dynamics of PTPN22 and CSK-mediated LCK regulation. CSK is recruited to the plasma membrane by binding to phosphorylated PAG through its SH2 domain. CSK can then phosphorylate LCK and inhibit it. Recruitment of CSK is inhibited by CD45-mediated dephosphorylation of PAG. Whether PTPN22 inhibits LCK activity when bound to CSK or when dissociated from it remains unclear. The mechanism by which PTPN22 is recruited to the plasma membrane to dephosphorylate LCK is currently unknown.

PTPN22 (also known as PEP in mice or LYP in humans) is also important for antigen discrimination, since cells that lack PTPN22 show increased T cell activation particularly in response to low affinity agonists (Salmond et al., 2014). PTPN22 interacts with CSK (Cloutier and Veillette, 1996), and this interaction is relevant for PTPN22 function, as shown by the fact that a human *PTPN22*^C1858T^ variant, encoding an amino acid R620W substitution which impairs its interaction with CSK (Bottini et al., 2004), is associated with increased risk of autoimmunity (Bottini et al., 2004; Totaro et al., 2011; de Lima et al., 2017; Tizaoui et al., 2019). However, whether PTPN22 inhibits TCR signaling more efficiently when interacting with or when dissociated from CSK remains unclear (Figure 3). In support of the latter, PTPN22 was shown to dissociate from cytosolic CSK and translocate to lipid rafts upon TCR stimulation, where it can access its substrates and inhibit TCR signaling (Vang et al., 2012).However, a mechanism for PTPN22 recruitment to and stabilization at the plasma membrane is missing. Another model, supported by a study using super-resolution imaging, suggests that interaction with CSK is induced upon integrin stimulation, and this interaction is important for driving PTPN22 to the plasma membrane and for downregulation of integrin signaling (Burn et al., 2016). Inducible interaction of PTPN22 and CSK upon TCR stimulation has also been reported (de la Puerta et al., 2013), but how the PTPN22-CSK complex would be recruited to and stabilized at the plasma membrane to reach its substrates is unclear. CSK reaches the plasma membrane because, via its SH2 domain, it binds phosphorylated Tyr on membrane adaptor proteins including phosphoprotein associated with glycosphingolipid-enriched microdomains (PAG) 1 (Davidson et al., 2003). However, the pool of CSK binding to PAG differs from the pool of CSK binding to PTPN22 (Davidson et al., 2016). Therefore, another mechanism would be needed to localize the PTPN22-CSK complex on the plasma membrane. The polyproline regions on PTPN22 allow interaction of this phosphatase with other proteins, hence other potential interaction partners could direct PTPN22 to the plasma membrane. Apart from CSK, the proline-serine-threonine phosphatase interacting protein 1 (PSTPIP1) is the only PTPN22 interaction partner identified so far (Voisinne et al., 2019). PSTPIP1 has been suggested to inhibit TCR signaling and localizes at the plasma membrane through its F-BAR and SH3 domains, interacting both with the cytoskeleton and with CD2 (Marcos et al., 2014). Further study of the interaction between PSTPIP1 and PTPN22 might help understanding the spatial regulation of PTPN22 in T cells, which is crucial to understand how the R620W polymorphism drives autoimmunity (further discussed in section “Tyr Phosphatases in T Cells”).

### Tyrosine Phosphatases Induce Amplification and Branching of Early TCR Signaling

Once TCR signaling is initiated by activation of SRC family kinases, it rapidly amplifies and branches to orchestrate the T cell response. Some of this branching is amplified by FYN, which induces amplification and diversification of TCR signaling by contributing to activation of the MAPK pathway (Lovatt et al., 2006) and by triggering cytoskeletal rearrangements downstream of the TCR (Chapman and Houtman, 2014). This is promoted by CD45 and receptor-type tyrosine-protein phosphatase α (RPTPα, encoded by the gene *PTPRA*), which activates FYN by dephosphorylating it on Y528 (Shiroo et al., 1992; Maksumova et al., 2007). In addition, dephosphorylation of Tyr on the adaptor protein PAG by a Tyr phosphatase, probably CD45, also sustains LCK and FYN activity (Davidson et al., 2003), since docking sites for CSK are lost upon PAG dephosphorylation.

#### LMPTP

LMPTP, encoded by the gene *ACP1*, positively regulates signaling downstream of the TCR by dephosphorylation of ZAP70 on the inhibitory Y292 (Bottini et al., 2002). This dephosphorylation prevents binding of the ubiquitin ligase c-CBL to ZAP70 and in consequence reduces ZAP70 degradation and prolongs TCR signaling. Although microscopy has shown that LMPTP localizes at the plasma membrane in lymphocytes (Gjorloff-Wingren et al., 2000), the mechanism of such localization remains unknown, since there is no obvious localization motif in its sequence, and no interaction partners have been identified. LMPTP is phosphorylated by SRC-family kinases on Tyr 131 and 132, and this increases its catalytic activity, generating a positive feedback loop for TCR signaling amplification (Tailor et al., 1997; Bucciantini et al., 1999).

### Tyrosine Phosphatases Drive Negative Feedback Loops and Signal Termination

Once downstream effectors of TCR signaling have been activated and the cellular response has been triggered, signaling must be terminated. Several Tyr phosphatases contribute to this process by dephosphorylation of SRC-family kinases, the ζ-chain and ZAP70.

SHP-1 has been proposed to contribute to signal termination by inhibition of LCK, since it is recruited to the TCR between 20 and 40 min after TCR stimulation with antigenic peptides (Stefanova et al., 2003). Recently, it was proposed that the thousand-and-one amino acid kinase 3 (TAOK3) is involved in the crosstalk between LCK and SHP-1 (Ormonde et al., 2018). Using the Jurkat cell line and anti-CD3 stimulation, the authors concluded that TAOK3 promotes TCR signaling by blocking LCK interaction with SHP-1. However, the only T cell phenotype of *TAOK3*^–/–^ mice reported so far was a reduction in CD8 T cell number (Hammad et al., 2017). A deeper analysis of T cell responses in these mice would help understand the relevance of TAOK3/SHP-1 crosstalk for T cell activation.

**FIGURE 4:**
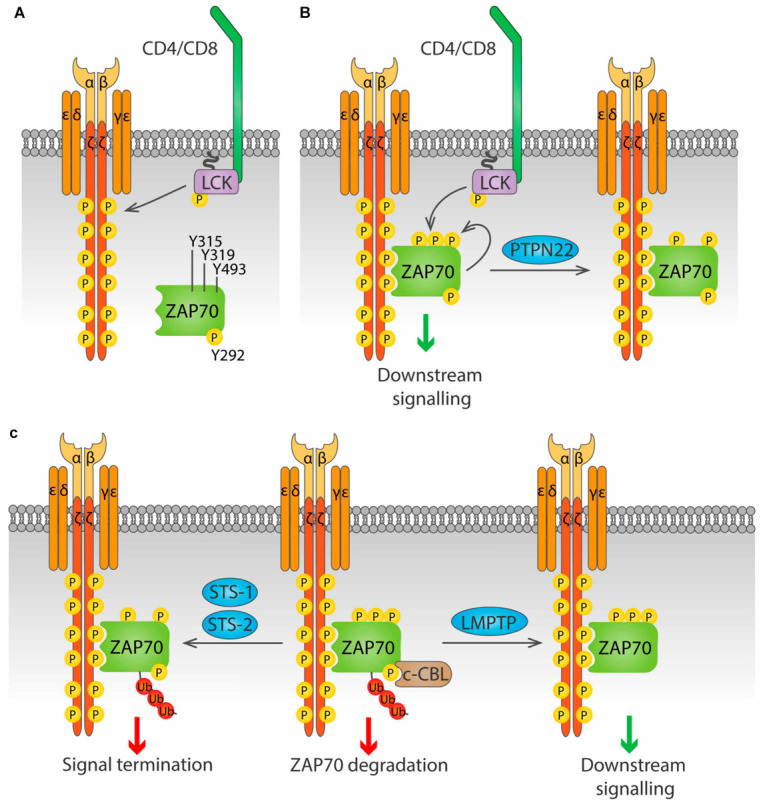
Regulation of ZAP70 by Tyr phosphatases. **(A)** ZAP70 is regulated by the phosphorylation status of three key residue, Y315, Y319 and Y493. Phosphorylation of the ζ chains by LCK upon antigen stimulation provides docking sites for ZAP70. **(B)** ZAP70 binds to phosphorylated ζ chains through its tandem SH2 domains. Binding to ζ chains induces a conformational change in ZAP70 that exposes Y315 and Y319, that can then be phosphorylated by LCK. Phosphorylation of Y493 either by LCK or by autophosphorylation leads to full activation of ZAP70. PTPN22 is able to dephosphorylate Y319, inhibiting ZAP70. **(C)** Phosphorylation on Y292 allows binding of the ubiquitin ligase c-Cbl to ZAP70, and subsequent addition of poly-ubiquitin that leads to ZAP70 degradation (center). This process is avoided by the Tyr phosphatase LMPTP, which dephosphorylates Y292 and blocks c-Cbl binding, prolonging ZAP70 signaling (right). The phosphatases Sts-1 and Sts-2 can bind ubiquitinated ZAP70 and dephosphorylate Y319, terminating ZAP70 signaling (right).

PTPN22 has been shown to dephosphorylate the ζ-chain both *in vitro* and in pervanadate-treated Jurkat cells (Wu et al., 2006). PTPN22 has also been suggested to dephosphorylate ZAP70 (Figure 4B). When a substrate-trapping PTPN22 mutant was expressed in Jurkat cells, ZAP70 was found among the bound proteins, and PTPN22 was shown to dephosphorylate ZAP70^pY319^ *in vitro* (Wu et al., 2006). Consistent with this observation, treatment of Jurkat cells with a PTPN22 inhibitor resulted in increased ZAP70 phosphorylation upon TCR stimulation (Vang et al., 2012). Evidence of direct dephosphorylation of these substrates in primary T cell is, however, not yet available.

The highly homologous non receptor phosphatases PTPN3 and PTPN4 (PTPH1 and PTP-MEG1, respectively) are both able to bind to and dephosphorylate the ζ-chain *in vitro* (Sozio et al., 2004; Young et al., 2008), and overexpression of either enzyme in Jurkat cells downmodulated T cell activation, although PTPN4 to a lesser extent (Han et al., 2000). However, none of the single knockout or the double *PTPN3*^–/–^*PTPN4*^–/–^ mice showed abnormalities in T cell activation or development (Bauler et al., 2008), suggesting that loss of these two phosphatases can either be compensated or lack relevance *in vivo*.

The two highly similar phosphatases STS-1 (also known as TULA-2, encoded by the *UBASH3B* gene) and STS-2 (also known as TULA, encoded by the *UBASH3A* gene) negatively regulate T cell activation through dephosphorylation of ZAP70^pY319^ (Carpino et al., 2004; Luis and Carpino, 2014) (Figure 4C). These phosphatases only bind to and dephosphorylate ubiquitinated ZAP70, providing a link between ubiquitination and phosphorylation-mediated regulation of early TCR signaling (Yang et al., 2015; Hu et al., 2016). Whether STS-1 and STS-2 are functionally redundant or have unique roles in T cell regulation remains largely unknown. Cells from STS-1^–/–^STS-2^–/–^ mice show increased T cell proliferation and cytokine production upon *in vitro* TCR stimulation compared to WT cells. In contrast, responses of T cells lacking only one STS member are only modestly increased, suggesting that these proteins are functionally redundant (San Luis et al., 2011). However, *in vivo* studies point to differential, although partially overlapping, roles of STS-1 and STS-2. Survival from systemic *Candida albicans* infection was significantly enhanced not only in STS-1^–/–^STS-2^–/–^ mice, but also in each single knockout mouse (Naseem et al., 2015). Similarly, lack of either phosphatase exacerbates pathology in a model of inflammatory bowel disease (IBD) (Newman et al., 2014). Nevertheless, in the latter study, only the double knockout mice showed enhanced cytokine production in the colon, and only double knockout CD4 T cells showed greater colitogenic capacity than wild type CD4 T cells when both were injected in T cell deficient, STS sufficient mice. The different outcomes are likely due to T cell-extrinsic effects of STS deficiency in the full knockout model used. Study of mice that lack STS-1, STS-2 or both specifically in the T cell compartment would help shed light into the specific functions of these proteins in T cell biology.

### Tyrosine Phosphatases Mediate Inhibitory Receptor Signaling

Several inhibitory receptors control T cell activation by inhibiting early TCR signaling (reviewed in Fuertes Marraco et al., 2015). This control is important to avoid T cell hyperactivation and damage derived from chronic antigen exposure. Inhibitory receptors lack intrinsic enzymatic activity but have cytoplasmic tails with immunoreceptor tyrosine-based inhibitory motifs (ITIMs) or an immunoreceptor tyrosine-based switch motif (ITSM) that are phosphorylated upon ligation and TCR signaling. The phosphorylated domains can serve as docking sites for the Tyr phosphatases with SH2 domains such as SHP-1 and SHP-2. This binding not only localizes SHP-1 and SHP-2 close to phosphorylated substrates, but also promotes a conformational change that leads to activation of the phosphatases (Hof et al., 1998; Wang et al., 2011). Below, we discuss the role of SHP-1 and SHP-2 in inhibition of early TCR signaling, and consequently T cell activation, downstream of several inhibitory receptors.

The role of SHP-1 and SHP-2 in signaling through the inhibitory receptor programmed cell death protein 1 (PD-1) is perhaps the most extensively studied, although it has been controversial. Initially, SHP-2, but not SHP-1, was shown to bind PD-1 upon PD-1 ligation, subsequently downregulating T cell activation through dephosphorylation of the ζ-chain and ZAP70 (Sheppard et al., 2004; Yokosuka et al., 2012). However, the finding that *SHP-2*^–/–^ mice show intact PD-1-mediated signaling and cell exhaustion (Rota et al., 2018) suggested that another phosphatase was recruited to PD-1, SHP-1 being the likely candidate. This controversy was recently resolved by Celis-Gutierrez and colleagues (Celis-Gutierrez et al., 2019). Using mass spectrometry, they defined the PD-1 interactome during PD-L1 ligation and antigen stimulation. They showed that in wild-type cells, SHP-2 is the main PD-1 interactor, binding 50 times more PD-1 molecules than SHP-1, despite the latter being approximately six times more abundant than SHP-2. In *SHP-2*^–/–^ cells, however, SHP-1 replaced SHP-2 and mediated PD-1 signaling. Consistently, only the double knockout *SHP-2*^–/–^*SHP-1*^–/–^ showed impaired PD-1-mediated T cell inhibition. This finding suggests that concomitant inhibition of both SHP-1 and SHP-2 would be needed to efficiently block PD-1 intracellular signaling in an immunotherapy setting. In the same study, the interactome of B and T lymphocyte attenuator (BTLA), another inhibitory receptor, was analyzed upon treatment of T cells with pervanadate (Celis-Gutierrez et al., 2019). Results showed that, consistent with a previous report (Watanabe et al., 2003), both SHP-1 and SHP-2 bind BTLA. However, contrary to PD-1, BTLA preferentially binds SHP-1 rather than SHP-2. This difference has implications for downstream inhibitory signaling. PD-1, recruiting mainly SHP-2, preferentially inhibits phosphorylation of CD28 over the ζ-chain, while BTLA, recruiting both SHP-1 and SHP-2, inhibits the phosphorylation of both CD28 and ζ-chain (Hui et al., 2017; Xu et al., 2020).

The role of SHP-1 and SHP-2 in cytotoxic T-lymphocyte antigen 4 (CTLA-4) signaling is poorly understood, with conflicting results being reported by different groups in the last 25 years. SHP-2 was initially shown to bind to CTLA-4 in T cells, although this binding would likely be indirect (Marengere et al., 1996; Schneider and Rudd, 2000). Supporting the need for an intermediate protein between CTLA-4 and SHP-2, another study did not find CTLA-4/SHP-2 association *in vitro* (Guntermann and Alexander, 2002; Yokosuka et al., 2010). Conflicting results are likely due to the different methodologies (immunoprecipitation *vs.* microscopy), cells (cell lines vs. primary murine cells), and conditions (*in vitro* proteins vs. cells; with endogenous *vs.* overexpressed proteins) used. Of note, CTLA-4 can exert inhibitory functions in a cell extrinsic manner and by signaling-independent mechanisms such as competition with CD28 for CD80/CD86 and transendocytosis of these ligands upon engagement (Walker and Sansom, 2015). Hence, the contribution of phosphatase-mediated signaling to CTLA-4 inhibition remains unclear. The study of the endogenous CTLA-4 interactome in primary T cells during antigen stimulation may help to identify whether there are cell intrinsic effects of CTLA-4 signaling and would be beneficial for applications in immunotherapy.

SHP-1 has been linked to signaling through two other inhibitory receptors, carcinoembryonic antigen-related cell adhesion molecule 1 (CEACAM1) and leucocyte-associated immunoglobulin receptor-1 (LAIR-1). Inhibition of T cell effector functions by CEACAM1 requires recruitment of SHP-1 (Nagaishi et al., 2006). During TCR stimulation, CEACAM1 ITIMs are phosphorylated by LCK, and serve as docking sites for SHP-1, which then dephosphorylates ZAP-70 and ζ-chain (Chen et al., 2008). On the other hand, SHP-1 constitutively interacts with LAIR-1 (Sathish et al., 2001a), a negative regulator of T cell activation highly expressed in naïve T cells (Maasho et al., 2005; Jansen et al., 2007). Although the relevance of this interaction for LAIR-1-mediated T cell inhibition has not been explored, it might be one of the mechanisms by which SHP-1 establishes T cell activation thresholds (Johnson et al., 1999; Sathish et al., 2001b).

Lastly, SHP-2, but not SHP-1, is recruited to platelet endothelial cell adhesion molecule-1 (PECAM-1, also known as CD31) (Newman et al., 2001), and ligation of PECAM-1 with agonist peptides during antigen presentation leads to SHP-2-dependent dephosphorylation of ZAP-70 and inhibition of T cell activation (Clement et al., 2015).

Altogether, regulation of T cell responses by inhibitory receptors strongly relies on SHP-1 and SHP-2, which makes these phosphatases attractive targets to enhance T cell responses. Despite a considerable improvement in the last years, more studies will be needed to clearly understand which functions are exclusive to SHP-1 or SHP-2, and in which situations loss of one of them can be compensated by the other. Strategies targeting both phosphatases are tempting, however their high expression and their regulatory role in important T cell functions such as cytokine signaling and adhesion will make it necessary to evaluate disruption of SHP-1/2 function for potential secondary effects.

## Tyrosine Phosphatases in Autoimmunity

Most T cell responses to pathogens are appropriately regulated, however approximately 4-5% of the population of developed countries suffers from an autoimmune disease (Hayter and Cook, 2012; Roberts and Erdei, 2020), the onset of which is generally considered to result from a failure of tolerance. In this context, it is striking that polymorphisms in genes encoding phosphatases are among the most frequently associated with autoimmune disease (Burton et al., 2007; Todd et al., 2007). Here, we will review the current evidence and understanding of several autoimmune diseases associated with PTPs and their aberrant expression (Table 2), and discuss what these diseases might tell us about the function of those PTPs.

**TABLE 2:** Tyr phosphatases associated with autoimmune diseases.

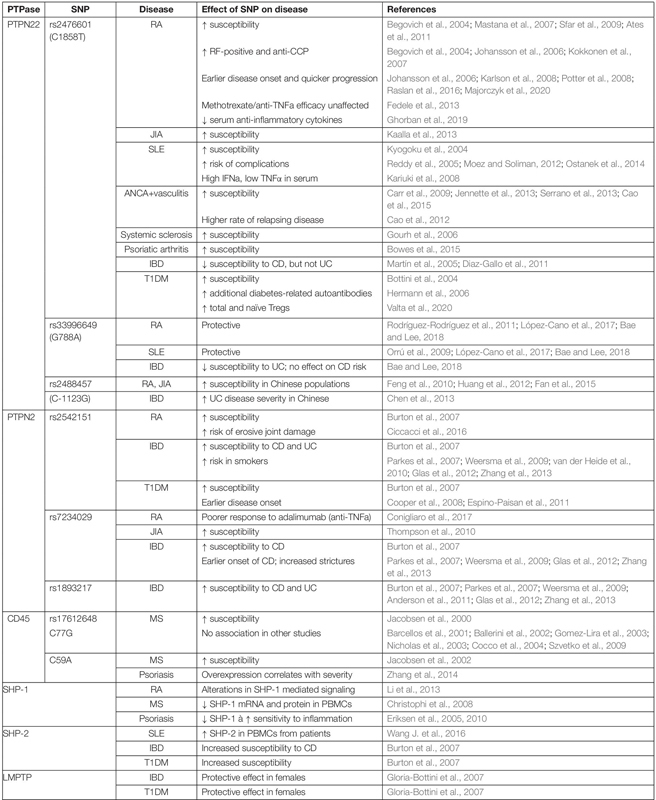

*RA, rheumatoid arthritis; RF, rheumatoid factor; CCP, cyclic citrullinated peptide; JIA, juvenile idiopathic arthritis; SLE, systemic lupus erythematosus; ANCA, anti-neutrophil cytoplasmic antibodies; IBD, inflammatory bowel disease; CD, Crohn’s disease; UC, ulcerative colitis; T1DM, Type 1 diabetes mellitus; MS, multiple sclerosis; PBMC, peripheral blood mononuclear cell.*

### Rheumatological Diseases

The rheumatological diseases are the archetype of autoimmune disease. This group of diseases is characterized by inflammation, predominantly affecting the joints, such as in rheumatoid arthritis (RA), but also connective tissues, such as in systemic sclerosis, and sometimes involving other specific organs, such as the skin, eye, mouth and serosae, as seen in systemic lupus erythematosus (SLE).

PTPN22 is the most extensively studied phosphatase in relation to autoimmune disease, and polymorphisms in the *PTPN22* gene are heavily associated with rheumatological diseases. The *PTPN22*^C1858T^ missense mutation (rs2476601), which leads to the R620W substitution, is the strongest non-HLA genetic association for autoimmune disease (Stanford and Bottini, 2014). In 2004, a significant association was first reported between the R620W variant and both RA (Begovich et al., 2004) and SLE (Kyogoku et al., 2004). These findings have subsequently been replicated numerous times, and *PTPN22*^C1858T^ has additionally been shown to be a risk factor for development of other rheumatological disease including ANCA-positive vasculitis (specifically microscopic polyangiitis, granulomatosis with polyangiitis, and giant cell arteritis, but not eosinophilic granulomatosis with polyangiitis) (Carr et al., 2009; Jennette et al., 2013; Serrano et al., 2013; Cao et al., 2015) and systemic sclerosis (Gourh et al., 2006). Northern European Caucasians are the most common carriers of this mutation, with a minor allele frequency of >10%, while individuals of Middle Eastern, Asian and African decent are more rarely affected (<1%) (Zheng et al., 2012). It is possible that this reflects a protective effect of the SNP against an infectious threat such as tuberculosis (Boechat et al., 2013). Despite this geographical variation, carriage of the *PTPN22*^C1858T^ SNP within populations with a lower minor allele frequency still appears to act as a susceptibility allele for RA (Mastana et al., 2007; Sfar et al., 2009; Ates et al., 2011).

The R620W mutation is not simply associated with RA, but has been shown to alter the pathogenesis and phenotype of the disease in patients with RA. Both homo- and heterozygosity for the *PTPN22*^C1858T^ allele are strongly associated with rheumatoid factor (RF)-positive disease (the presence of circulating antibodies), while RF-negative disease shows no association (Begovich et al., 2004; Kokkonen et al., 2007). Furthermore, the C1858T variant is strongly associated with the additional presence of anti-cyclic citrullinated (anti-CCP) antibodies (Johansson et al., 2006; Kokkonen et al., 2007), earlier disease onset (Johansson et al., 2006; Karlson et al., 2008), quicker progression of radiological joint destruction (Lie et al., 2007), and erosive disease (Raslan et al., 2016). Interestingly, the presence of the *PTPN22*^C1858T^ SNP has no effect on the efficacy of anti-TNFα drug treatments used in RA (Potter et al., 2008), and studies examining its effect on efficacy of methotrexate have similarly shown mixed results without a convincing effect (Fedele et al., 2013; Majorczyk et al., 2020).

The effect of the *PTPN22*^C1858T^ SNP is not confined to T cells, but also involves B cells and myeloid cells, although detailed description of their involvement is outside the scope of this review. In T cells, an early study demonstrated that T cells from human donors heterozygous for the R620W variant secreted significantly less IL-2 in response to TCR stimulation (Vang et al., 2005). Several subsequent studies demonstrated reduced calcium mobilization and CD25 expression in response to TCR stimulation in C1858T homozygous human CD4 T cells (Rieck et al., 2007), resulting in reduced T cell proliferation (Aarnisalo et al., 2008) and IL-2 production (Aarnisalo et al., 2008; Chuang et al., 2009). T cells from healthy human homozygotes without clinically apparent autoimmune disease demonstrated reduced ζ-chain phosphorylation in response to TCR stimulation, due to increased phosphatase activity (Vang et al., 2013).

At a cellular level, the outcome of these alterations in signaling appears to be a shift towards a pro-inflammatory state lacking autoimmunity-protective mechanisms. Patients with SLE carrying the *PTPN22*^C1858T^ risk allele show a skewing towards high serum IFNα and low TNFα compared with patients without the SNP (Kariuki et al., 2008), a profile that has been implicated as a risk factor for SLE (Niewold et al., 2007). Furthermore, circulating levels of anti-inflammatory cytokines such as IL-10 have been shown to be reduced in individuals with RA carrying the *PTPN22*^C1858T^ SNP (Ghorban et al., 2019). Reduced IL-10 mRNA expression was also demonstrated in heterozygous patients with ANCA-positive vasculitis, due to high basal PTPN22 phosphatase activity conferring decreased phosphorylation of ERK; this correlated clinically with a higher rate of relapsing disease (Cao et al., 2012). In T cells from healthy human donors homozygous for *PTPN22*^C1858T^, CD4 T cells produced significantly more IFNγ compared to those from individuals without the mutant allele, and significantly less IL-17, suggesting a skew in CD4 T cell differentiation away from Th17 towards Th1 (Vang et al., 2013). Additionally, CD4^+^Foxp3^+^ regulatory T cells (Treg) appear to be altered in the presence of the SNP. In chimeric mice reconstituted 1:1 with WT and PTPN22 R619W (the murine equivalent of R620W) bone marrow, more Tregs carrying the R619W mutation developed, indicating that PTPN22 exerts a cell intrinsic bias towards development of this lineage (Knipper et al., 2020). In *PTPN22*^C1858T^ carriers with type 1 diabetes, higher frequencies of total and naïve Tregs have been seen, suggesting that in humans also PTPN22 exerts an effect on circulating numbers of these cells (Valta et al., 2020). Furthermore, Tregs from C1858T homozygous human donors were not able to suppress the secretion of IFNγ by conventional CD4 T cells, suggesting the balance between regulatory and effector/memory cells is disrupted in such individuals (Vang et al., 2013).

The *PTPN22*^C1858T^ variant is also associated with juvenile idiopathic arthritic (JIA), and notably this association has been demonstrated by meta-analysis to be strongest with the RF-positive polyarticular JIA subtype, which is most similar to RA (Kaalla et al., 2013). Furthermore, susceptibility to ANCA (anti-neutrophil cytoplasmic antibody)-positive vasculitis is increased in the presence of the R620W allele, and specifically to involvement of lung, skin, ear/nose/throat, and peripheral neuropathy (Cao et al., 2015). Another rheumatological disease associated with the *PTPN22*^C1858T^ SNP is SLE, in which homozygosity poses a much higher risk (OR 4.37, vs. 1.37 for heterozygotes) (Kyogoku et al., 2004). Similarly to RA, the presence of the SNP not only confers increased susceptibility to SLE, but may also alter its clinical course: carriage of *PTPN22*^C1858T^ is associated with increased risk of renal complications of SLE (Reddy et al., 2005; Moez and Soliman, 2012), as well as secondary antiphospholipid syndrome (Ostanek et al., 2014). Higher titers of anti-cardiolipin and lupus anticoagulant antibodies were also found in SLE patients carrying *PTPN22*^C1858T^ (Ostanek et al., 2014). These associations illustrate the fact that C1858T is predominantly linked to autoimmune diseases characterized by the presence of circulating auto-antibodies (Begovich et al., 2004; Padyukov et al., 2011; Zheng et al., 2012), and suggests that pathogenic B cells play a role in R620W-associated disease. Although the role of PTPN22 in B cell receptor signaling is less well defined, human B cell activation is inhibited by the C1858T polymorphism, suggesting that impaired elimination of autoreactive B cells may be a factor (Menard et al., 2011; Metzler et al., 2017). Given the evidence of T cell influence in *PTPN22*^C1858T^ associated diseases, it is likely that follicular helper T cells (Tfh), which are essential for B cell responses in the germinal centers, are relevant. This seems to be the case in mice at least, where knockout of *Ptpn22* led to increased Tfh proliferation and accumulation in the germinal centers, as well as enhanced IL-21 production (Maine et al., 2014), while in non-obese diabetic (NOD) mice expressing the R619W variant there were increased Tfh and germinal center B cell numbers, associated with increased anti-islet auto-antibodies (Schmiel et al., 2018).

In addition to C1858T, other *PTPN22* polymorphisms have been identified, although none are as frequent nor as widely studied. The G788A missense mutation (rs33996649) causes a substitution of arginine to glutamine at position 263 (R263Q), located in the catalytic domain. This results in a change in conformation at the active site, manifesting as reduced phosphatase activity (Orrú et al., 2009). Despite conferring loss-of-function, G788A has been shown to be protective against RA (Rodríguez-Rodríguez et al., 2011; López-Cano et al., 2017; Bae and Lee, 2018) and SLE (Orrú et al., 2009; López-Cano et al., 2017; Bae and Lee, 2018). The *PTPN22*^C–1123G^ SNP has also been linked to a higher risk of RA and JIA, but only affecting Chinese individuals (Feng et al., 2010; Huang et al., 2012; Fan et al., 2015); in Caucasian populations it was not demonstrated to increase risk of RA independently of C1858T, with which it is often co-expressed (Dieudé et al., 2008).

A further ubiquitously expressed phosphatase, PTPN2, has also been linked to RA (Burton et al., 2007) and JIA (Thompson et al., 2010), as well as other autoimmune diseases to be discussed in more detail later in this section. Similarly to PTPN22, SNPs in the *PTPN2* gene have been shown to confer specific disease phenotypes and/or response to therapies. For example, the rs2542151 SNP is associated with higher risk of erosive joint damage in RA patients (Ciccacci et al., 2016). Furthermore, the rs7234029 SNP has been linked to poorer response to treatment of RA with adalimumab (Conigliaro et al., 2017), an anti-TNFα monoclonal antibody.

Further T cell PTPs that have been implicated in RA and other rheumatological diseases include SHP-1 and SHP-2. Administration of the SHP-1 agonist regorafenib to mice with inflammatory arthritis significantly decreased incidence and severity of joint inflammation via increased phosphatase activity and decreased IFNγ secretion by splenic T cells (Markovics et al., 2020). However, the effects of SHP-1 dysregulation are not limited to T cells, due to its widespread expression in all hematopoietic cells as well as epithelial cells (Lorenz, 2009). In rheumatoid arthritis, inflammation associated with alterations in SHP-1-mediated signaling are mediated through T cells, B cells and macrophages (Li et al., 2013), while deletion of SHP-1 in B cells in mice causes an SLE-like disease (Pao et al., 2007). With regards to SHP-2, SHP-2 activity is higher in PBMCs from patients with SLE than from healthy individuals, and SHP-2 inhibition has been shown to significantly reduce T cell proliferation and production of IFNγ and IL-17 (Wang J. et al., 2016). Analogously, lupus prone mice treated with a SHP-2 inhibitor exhibited less severe disease (Wang J. et al., 2016).

### Inflammatory Bowel Disease

Inflammatory bowel disease (IBD) is an umbrella term for ulcerative colitis (UC) and Crohn’s disease (CD), which are characterized by chronic inflammation in the gastrointestinal tract, leading to symptoms of abdominal pain, diarrhea, rectal bleeding, weight loss and fatigue. An acute severe flare may lead to complications such as toxic megacolon or bowel perforation, while long term inflammation can cause severe ulceration, abscesses and bowel strictures.

Polymorphisms in the *PTPN2* gene have been heavily linked with several autoimmune diseases including IBD (Glas et al., 2012; Zhang et al., 2013). There are several SNPs that have been identified by genome wide association studies (GWAS) as being associated with IBD: rs2542151 (located 5.5 kb upstream from the PTPN2 gene), rs7234029, and rs1893217 (Glas et al., 2012; Zhang et al., 2013). All three SNPs are associated with CD (Burton et al., 2007; Parkes et al., 2007; Weersma et al., 2009; Glas et al., 2012; Zhang et al., 2013), while rs2542151 (Anderson et al., 2011; Glas et al., 2012; Zhang et al., 2013), and rs1893217 (Anderson et al., 2011) are also associated with UC. As well as conferring susceptibility to IBD, the presence of the rs7234029 correlates with a stricturing disease phenotype and earlier onset of CD (Glas et al., 2012). Interestingly, a recent meta-analysis of 13 studies showed differences between ethnicities, with rs2542151 increasing risk of both CD and UC in Caucasian but not in Asian study populations (Zhang et al., 2013). Furthermore, a study investigating the differences in genetic background between smoking and non-smoking Dutch-Belgian patients with Crohn’s disease found that the rs2542151 *PTPN2* SNP only increased susceptibility in the smoking cohort, but not in the non-smoking or complete cohort (van der Heide et al., 2010).

IBD is characterized by loss of tolerance to intestinal commensal bacterial and self-antigens, due to dysregulated CD4 T cell differentiation, with enhanced differentiation of Th1 and Th17 cells, as demonstrated by elevated levels of IFNγ, IL-17, and IL-22 in the intestinal biopsies and serum of patients with IBD (Fujino et al., 2003; Maloy and Powrie, 2011). In mouse models of colitis, T cell-specific loss of PTPN2 leads to increased numbers of Th1 and Th17 cells in the colonic lamina propria, mesenteric lymph nodes and spleen, corresponding with earlier onset and increased severity of disease (Spalinger et al., 2015). Mirroring this, humans with IBD carrying the PTPN2 rs1893217 SNP have greater Th1- and Th17-associated gene expression in colonic biopsies (Spalinger et al., 2015). Furthermore, there is impaired induction of regulatory T cells (Treg) in PTPN2 deficient colitic mice compared to PTPN2 competent counterparts (Spalinger et al., 2015). A recent study using a *Ptpn2* haplo-insufficient auto-inflammatory mouse model demonstrated that reduced PTPN2 expression (as occurs in human carriers of *PTPN2* SNPs) led to increased disease severity, mediated through a Treg intrinsic mechanism in which PTPN2 dephosphorylation of STAT3 prevents pathogenic loss of FoxP3 after acquisition of RORγt by Tregs (Svensson et al., 2019). However, this mouse model expresses very little ZAP-70, so the outcome may differ from otherwise normally signaling cells lacking PTPN2. These results are also somewhat conflicting with previous studies suggesting that loss of PTPN2 enhanced Treg number and/or function (Wiede et al., 2011; Yi et al., 2014; Bothur et al., 2015) so the influence of PTPN2 on Treg differentiation may depend on the inflammatory environment present in the different autoimmune models. In CD8 T cells, PTPN2 deficiency induces enhanced thymic positive selection and accumulation of peripheral effector/memory T cells, leading to systemic autoinflammatory disease, which was reproducible in wild-type recipient mice following adoptive transfer of CD8 T cells (Wiede et al., 2011).

In addition to its interaction with LCK and FYN, PTPN2 is also known to negatively regulate JAK/STAT pathways (Simoncic et al., 2002; ten Hoeve et al., 2002). JAK/STATs mediate signaling through receptors for inflammatory cytokines such as IL-2 and IFNγ (Simoncic et al., 2002), as well as cytokines, such as IL-7, that direct T cell differentiation and homeostasis (Pike et al., 2017). PTPN2 may also regulate the T cell repertoire by controlling thymocyte lineage commitment and TCR specification through both LCK and STAT5 dephosphorylation (Wiede et al., 2017a). Thus, PTPN2 downregulates T cell activation and differentiation/development through two independent mechanisms. However, the postulated effect of PTPN2 on JAK/STAT signaling has been challenged by the finding that a *PTPN2* risk allele (rs1893217) correlated with reduced PTPN2 expression and reduced (rather than increased, as might be expected) phosphorylated STAT5 in response to IL-2 and IL-15 (Long et al., 2011), highlighting its probable complex action in multiple cell lineages.

It is important to note that, like PTPN22, the action of PTPN2 is not confined to the T cell compartment. This is demonstrated by the differences in disease phenotypes between mice that are completely deficient in PTPN2 and those with conditional deletion in T cells alone. In the former, autoimmune disease is more severe and occurs at a much earlier stage of life (You-Ten et al., 1997; Heinonen et al., 2004; Wiede et al., 2017b), confirming that PTPN2 plays an essential role in other cell types of both the innate and adaptive immune system to prevent autoimmunity. Moreover, PTPN2 is also expressed in tissues out with the hematopoietic system, and it is likely that its role in autoimmune disease is mediated through these as well. For example, PTPN2 is also expressed in intestinal epithelial cells, where it modulates cytokine secretion in response to TNFα and regulates epithelial permeability (Scharl et al., 2009, 2011).

Polymorphisms in the *PTPN22* gene are also associated with IBD, although the different SNPs differ in their effect. Interestingly, the classical C1858T SNP does not have any effect on risk of UC (Martín et al., 2005), while the rarer SNPs G788A and C-1123G do: the former reduces the risk of UC (Bae and Lee, 2018), while in Chinese populations the latter increases UC disease severity (Chen et al., 2013). Conversely, PTPN22 C1858T reduces the risk of CD, while G788A has no effect on CD risk (Diaz-Gallo et al., 2011; Bae and Lee, 2018).

### Type 1 Diabetes Mellitus

Diabetes mellitus is a metabolic disorder characterized by absence of pancreatic insulin secretion (type 1) or lack of peripheral response to insulin (type 2), leading to elevated blood glucose levels and, if untreated, macro- and microvascular complications such as ischemic heart disease, stroke, peripheral neuropathy, nephropathy, and retinopathy. Type 1 diabetes (T1DM) is an autoimmune disease caused by antibody-mediated destruction of insulin producing beta cells in pancreatic islets of Langerhans that usually manifests during childhood or adolescence and persists lifelong.

Increased risk of T1DM has been linked to SNPs in both of the phosphatases already discussed, PTPN22 (Bottini et al., 2004) and PTPN2 (Burton et al., 2007; Cooper et al., 2008; Espino-Paisan et al., 2011). In children with risk-associated HLA genotypes, carriage of the PTPN22 R620W SNP is associated with earlier onset of clinical T1DM, reflected in earlier appearance of islet auto-antibodies, as well as a higher likelihood of developing additional diabetes-associated auto-antibodies such as glutamic acid decarboxylase autoantibodies and islet antigen-2 autoantibodies (Hermann et al., 2006). Similarly, *PTPN2* polymorphisms are associated with earlier onset of disease (Espino-Paisan et al., 2011). This is backed up by mouse models, in which adoptive transfer of PTPN2-deficient CD8 T cells resulted in beta cell destruction and development of autoimmune diabetes, and this was exacerbated by co-transfer of PTPN2-deficient CD4 T cells (Wiede et al., 2014). Recently, novel mutations in coding regions of *PTPN2* were identified as susceptibility factors for development of childhood-onset T1DM in a Japanese population (Okuno et al., 2018), but these findings are yet to be replicated more widely.

Again, it is noteworthy that expression of PTPN22 and PTPN2 is not confined to T cells: PTPN22 expression is restricted to all hematopoietic cells, while PTPN2 is expressed more ubiquitously. Thus, the effects of their relevant SNPs on predisposition to autoimmune diseases are not mediated solely through T cells. For example, PTPN2 regulates cytokine-induced pancreatic β cell apoptosis (Moore et al., 2009), β cell insulin secretion (Xi et al., 2015), and insulin receptor signaling in muscle and liver (Galic et al., 2003), all of which contribute to T1DM pathogenesis. To attempt to determine the effect of PTPN2 deficiency in T cells specifically, Wiede et al. recently utilized a NOD mouse model (in which autoimmune diabetes occurs spontaneously) in which PTPN2 was lacking only in T cells. Their results demonstrated that T cell specific deficiency of PTPN2 led to increased incidence and earlier onset of autoimmune diabetes (Wiede et al., 2019). This was associated with pancreatic islet infiltration by CD8 and Th1 cells, as well as expansion of Tfh and B cells in the spleens, inguinal lymph nodes, and pancreatic draining lymph nodes, reinforcing the role for auto-antibodies in the disease pathogenesis (Wiede et al., 2019).

Mutations in the *PTPN11* gene (encoding SHP-2) are also associated with increased risk of T1DM (Burton et al., 2007), while an *ACP-1* (encoding LMPTP) polymorphism reduces risk. The latter association is subtler and appears to influence Th1/Th2 orientation depending on gender. The presence of the ACP1^∗^A allele, which leads to low LMPTP activity, increases female susceptibility to allergic disorders (Th2-mediated), while reducing female susceptibility to T1DM and Crohn’s (Th1-mediated) compared to males (Gloria-Bottini et al., 2007). However, the mechanism behind this may lie outside of T cells: in diabetes, at least, LMPTP appears to be a key promoter of insulin resistance through its dephosphorylation of the insulin receptor in the liver (Stanford et al., 2017).

### Multiple Sclerosis

Multiple sclerosis (MS) is a chronic inflammatory disease of the central nervous system (CNS) caused by autoimmune neuronal demyelination leading to signal conduction block or slowing. The symptoms can be variable due to the potential for the disease to affect any part of the CNS; patients may experience some recovery between episodes (relapsing-remitting MS, the most common form) or there may be no remission phase (primary and secondary progressive MS). In the majority of cases, the disease is progressive, with accumulation of neurological deficits over time, and it is one of the leading causes of disability in the developed world. In contrast to the previously discussed antibody-mediated autoimmune diseases, MS is classically driven by CNS-infiltrating T lymphocytes causing destruction of the myelin sheath and the oligodendrocytes that produce it, in response to myelin antigens. Correspondingly, the T cell tyrosine phosphatases implicated in this disease are distinct from those discussed in the previous sections. Indeed, *PTPN22*^C1858T^ shows no correlation with MS risk. The notion of MS being a purely T cell driven disease has been challenged somewhat recently by the success of anti-CD20 monoclonal antibody treatments for MS (Bar-Or et al., 2008; Hauser et al., 2009, 2017), revealing an important role for B cells in the pathogenesis. However, it is thought that these pathogenic B cells play more of a role in antigen presentation and T cell activation rather than antibody production (Jelcic et al., 2018), and the autoreactive T cell remains the central player in MS.

Mutations in the *PTPRC* gene, encoding CD45, are associated with MS. Different highly conserved isoforms of CD45 may be expressed due to alternative splicing of exons 4, 5 and 6, giving rise to CD45RA, RB, and RC, respectively (Trowbridge and Thomas, 1994; Pulido et al., 1988). Different isoforms are expressed at distinct stages of T cell development (for example CD45RB on naïve cells; CD45RO on activated and memory cells) (Clement, 1992), and they differ in their ability to modulate TCR signaling. This has been suggested to be related to their relative size, which influences their ability to form homodimers (Xu and Weiss, 2002), as well as the speed and efficiency with which CD45 may be excluded from the TCR-pMHC complex in the immunological synapse to reduce local phosphatase activity, enhancing phosphorylation and TCR signaling (Leupin et al., 2000; Davis and van der Merwe, 2006; Cordoba et al., 2013; Carbone et al., 2017). A C77G point mutation, which prevents silencing of exon 4 splicing, leading to overexpression of the CD45RA isoform in T cells (Thude et al., 1995; Lynch and Weiss, 2001), has been described at greater frequency in patients with MS compared to healthy controls (Jacobsen et al., 2000). The alteration in isoform expression has been suggested to lead to reduced dimerization and autoinhibition of CD45, thereby enhancing CD45 phosphatase activity. T cells from heterozygous healthy human donors and patients with MS demonstrated increased proliferation and IL-2 production in response to TCR ligation (Do et al., 2006). A similarly enhanced proliferation was seen in response to stimulation with IL-2 (Windhagen et al., 2007). In addition, Tregs from C77G carriers showed impaired responsiveness to TCR/CD28 stimulation and reduced ability to suppress conventional CD4 T cells (Pokoyski et al., 2015). However, the association between the C77G SNP and MS has only been corroborated by some subsequent studies (Ballerini et al., 2002) but not others (Barcellos et al., 2001; Gomez-Lira et al., 2003; Nicholas et al., 2003; Cocco et al., 2004; Szvetko et al., 2009), although this disparity may be because of the case-control design of most primary studies and low allelic frequency in most populations (Tchilian and Beverley, 2006). It has furthermore been argued that any potential role played by CD45 in MS may actually relate to its function in oligodendrocyte development and myelination in the CNS (Nakahara et al., 2005). A further human CD45 polymorphism, C59A, alters alternative splicing, leading to expression of CD45RA on memory T cells and monocytes, and has been linked to MS in one MS multiplex family (Jacobsen et al., 2002).

In mice, a single point mutation in the CD45 wedge motif, glutamate 613 to arginine (E613R), prevents the formation of CD45 dimers, and negative regulation of CD45 is lost, leading to development of lymphoproliferative disease and severe autoimmune lupus-like nephritis (Majeti et al., 2000). Thymocytes from these mice exhibit enhanced TCR-induced MAPK activation and calcium flux, undergo positive selection more readily, and have higher numbers of peripheral T cells. These mice are more sensitive to experimental autoimmune encephalomyelitis (EAE) (Hermiston et al., 2009), a Th1 cell driven inflammatory demyelinating disorder of the central nervous system (CNS) frequently used as a mouse model of MS.

Alterations in SHP-1 signaling are also associated with MS, as well as other autoimmune diseases. So-called “motheaten” mice have a recessive *Ptpn6* frameshift mutation that leads to an absence of SHP-1 protein (Green and Shultz, 1975; Shultz et al., 1993; Tsui et al., 1993), and exhibit severe skin inflammation, as well as interstitial pneumonitis and a range of hematological abnormalities, including hyperproliferative T cells (Minton, 2013). PBMCs from patients with MS have reduced levels of SHP-1 mRNA and protein (Christophi et al., 2008), due to increased DNA methylation of the *SHP-1* promoter (Kumagai et al., 2012). This acquired deficiency of SHP-1 is thought to lead to T cell induced inflammation through a reduction in dephosphorylation of targets such as STAT1, STAT6, NFκB and consequent increase in STAT-responsive inflammatory genes (Feng et al., 2002; Christophi et al., 2009). Furthermore, treatment of PBMCs from MS patients with IFNβ (a current treatment for MS) induces SHP-1 activity with corresponding reduced inflammatory gene expression, and the therapeutic effect of IFNβ is also dependent on SHP-1 (Christophi et al., 2009). This is backed up by EAE mouse models, in which heterozygous deletion of SHP-1 led to increased IFNγ production and increased expansion of MBP (myelin basic protein, the predominant auto-antigen) specific T cells in response to lower antigen concentrations, and these mice developed a more severe EAE phenotype (Deng et al., 2002). However, acquired deficiency of SHP-1 is not likely to be a direct cause of MS, rather it confers susceptibility to auto-inflammatory demyelination if other conditions are met, as has been demonstrated in mice (Croker et al., 2008). In addition, while T cells play a significant role in the pathogenesis of MS, the effects of SHP-1 deficiency in other cells types such as myeloid cells and oligodendrocytes is also expected to be important (Gruber et al., 2015).

SHP-2 may also participate in T cell driven pathology in MS, as treatment of mice with a SHP-2 inhibitor enabled resistance to induction of EAE following inoculation with myelin oligodendrocyte glycoprotein35-55 (MOG) protein, via prevention of infiltration of CD8 T cells into the CNS (Luo et al., 2014). These observations are yet to be borne out in human studies, where the picture is likely to be more complicated.

### Psoriasis

Auto-reactive T cells also play a central role in psoriasis, a chronic relapsing inflammatory skin disease characterized by epidermal hyperplasia and desquamation. Specifically, epidermal CD8 T cells that respond to skin epitopes mediate the initiation phase of the disease (Johnston et al., 2004; Lande et al., 2014; Arakawa et al., 2015), and subsequent amplification of skin inflammation is driven by a predominantly Th17 response (Lowes et al., 2013; Girolomoni et al., 2017). The central importance of the Th17 axis has been highlighted by recent success of anti-IL-17 monoclonal antibodies in the treatment of psoriasis (Mease et al., 2014; McInnes et al., 2015).

Aberrations in the same phosphatases as those linked to MS are also associated with psoriasis. T cells from patients with psoriasis are more sensitive to IFNα-induced stimulation, leading to increased STAT signaling and pro-inflammatory IFNγ production (Eriksen et al., 2005). This has been shown to be mediated through reduced expression of SHP-1 in psoriatic T cells, and was reversible by the forced expression of SHP-1 in T cells from the skin of psoriasis patients (Eriksen et al., 2010). In contrast to MS, in psoriasis the reduction in SHP-1 is due to demethylation of the promotor 2 of the gene (Ruchusatsawat et al., 2006).

CD45 has been shown to be significantly overexpressed in the bone marrow hematopoietic stem cells and PBMCs of patients with psoriasis, compared to those from healthy controls (Zhang et al., 2014). This higher level of CD45 expression correlated with disease severity index (Zhang et al., 2014), suggesting that this could be used as a biomarker for severity.

Interestingly, while the PTPN22 R620W polymorphism does not associate with skin psoriasis, it does increase the risk of psoriatic arthritis (Bowes et al., 2015), suggesting that the two diseases have diverging pathogeneses, and PTPN22 may in some way alter the balance or phenotype of CD8 and/or Th17 cells, particularly when the known action of PTPN22 on CD4 T cell differentiation is taken into consideration (Vang et al., 2013).

### Other Autoimmune Diseases Associated With T Cell PTPs

There are several other autoimmune diseases that have been linked to PTP mutations or altered expression. *PTPN22*^C1858T^ is the predominant association, and has been linked to Grave’s disease (Velaga et al., 2004; Heward et al., 2007), vitiligo (Cantón et al., 2005), myasthenia gravis (MG) (Vandiedonck et al., 2006; Chuang et al., 2009), Addison’s disease (Skinningsrud et al., 2008), and alopecia areata (Lei et al., 2019). Grave’s disease (autoimmune-mediated hyperthyroidism) is also associated with polymorphisms in the *PTPN2* gene (Todd et al., 2007).

The tyrosine phosphatases discussed here are those most studied with respect to autoimmune disease, but the list is not exhaustive. Although several human PTP SNPs have been linked to autoimmunity through GWAS, there is still much work to be done in order to deepen our understanding of the immunopathogenic mechanisms. It is striking that diseases that are strongly auto-antibody mediated, such as most rheumatological diseases, are affected by alterations in PTPN22 and PTPN2, whereas T cell driven diseases such as MS and psoriasis lean more heavily towards changes in other PTPs such as CD45 and SHP-1. This may suggest that the different PTPs influence different types of immune response, or be due to the relative influence of each PTP on different populations of T cells, for example increased Th1 and Tfh responses, compared with enhanced CD8 and Th17 functions. Autoimmune diseases are polygenic, and it is likely that an individual PTP mutation confers only modest relative risk of developing disease; rather disease occurs in the context of complex genetic and environmental pre-disposing factors. Deciphering the relative contributions to disease of individual PTPs and interrogating them as potential therapeutic targets should be a focus for future work.

## Tyrosine Phosphatases as Targets in Immunotherapy

**FIGURE 5:**
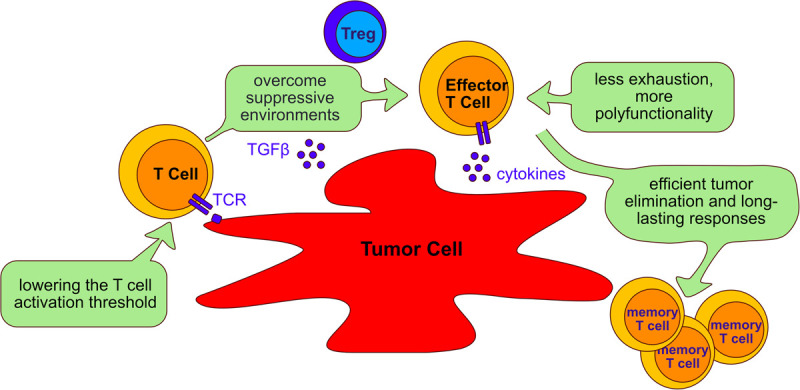
Aims of adoptive cell transfer therapy for treatment of cancer. Adoptive cell transfer strategies aim to: (I) lower the T cell activation threshold to allow response to tumor antigens; (II) overcome the suppressive environment generated by tumor cells and immunosuppressive cells such as Tregs; (III) reduce T cell exhaustion and increasing polyfunctionality of T cells; (IV) enable long lasting responses that allow persistent tumor elimination. The beneficial role of targeting the phosphatases PTPN2, PTPN22, SHP-1 and SHP-2 to improve adoptive cell transfer therapy is discussed in the text (Section “Tyrosine phosphatases as targets in immunotherapy”).

Immunotherapy is the use of the immune system to fight cancer. There are different kinds of immunotherapy, for instance, monoclonal antibodies that target inhibitory molecules like PD-1 and CTLA-4 are called checkpoint inhibitors. Additionally, tumor antigens can be used to target cancer cells. Adoptive T cell therapy (ACT) is a novel modality of immunotherapy using either tumor infiltrating lymphocytes (TILs) from the patient or engineered T cells with a TCR or a chimeric antigen receptor (CAR) that recognizes tumor antigens. Both options can induce complete and durable regression of tumors (Johnson et al., 2009; Rosenberg et al., 2011; Robbins et al., 2015). Despite the successful treatment of a proportion of cancer patients with ACT, the majority of patients do not yet benefit from the therapy, especially when treating solid tumors. The challenges faced by adoptively transferred T cells in eliminating tumors is illustrated in Figure 5 and below we discuss studies that have targeted four phosphatases, PTPN2, PTPN22, SHP-1 and SHP-2 as a strategy of overcoming these hurdles and improving ACT in several cancer models (Table 3).

**TABLE 3:** Functional outcomes of Tyr phosphatase deletion in T cells.

**PTPase**	**KO Mouse**	**Functional Outcome**	**References**
PTPN2	pLck-Cre; PTPN2^fl/fl^	↑ effector/memory T cells	Wiede et al., 2011
	Mx1-Cre; PTPN2^fl/fl^		Wiede et al., 2017b
	pLck-Cre; PTPN2^fl/fl^ OT-1	↑ response to low affinity ligands	Wiede et al., 2011
	PTPN2 sgRNA/Cas9 OT-1	↑ response B16-OVA	LaFleur et al., 2019
	Lck-Cre; PTPN2^fl/fl^ HER2 CAR	↑ PD-1 and LAG-3 expression	Wiede et al., 2020
	CD4-Cre; PTPN2^fl/fl^	↑ Th1 and Th17, ↓ Treg	Spalinger et al., 2015
PTPN22	PTPN22^–/–^ Rag1^–/–^ OT-1	↑ effector/memory T cells	Hasegawa et al., 2004; Brownlie et al., 2012
		↑ response to low affinity ligands & tumors	Salmond et al., 2014; Brownlie et al., 2017, 2019
		↑ polyfunctionality	Salmond et al., 2014
		↑ resistance to suppressive cytokines and Tregs	Brownlie et al., 2017
		↑ proliferation	Knipper et al., 2020
	PTPN22 sgRNA/Cas9	↑ response to tumors	Cubas et al., 2020
	PC3-Cre; PTPN2^fl/fl^ OT-1	↑ Treg suppression & IL-10 secretion	Brownlie et al., 2012
	PTPN22^–/–^	↑ Treg	Maine et al., 2012; Knipper et al., 2020
SHP-1	Lck-Cre; SHP-1^fl/fl^	↑ CD8 T cells proliferation	Fowler et al., 2010; Stromnes et al., 2012
		↑ polyfunctionality	Stromnes et al., 2012
		↑ response to leukemia cells	Stromnes et al., 2012
		↑ resistance to Tregs	Mercadante and Lorenz, 2017
		↓ short-lived effector cell formation	Fowler et al., 2010
	shRNA KD OT-1	↑ polyfunctionality	Snook et al., 2020
	SHP-1 sgRNA/Cas9 CD19 CAR	↑ polyfunctionality	Ruella et al., 2020
		↑ response to tumors	Ruella et al., 2020
	Motheaten mutant	↑ IL-2 production in CD4 T cells, ↓ requirement for CD28 co-stimulation	Sathish et al., 2001b
		↑ Treg suppression	Iype et al., 2010
SHP-2	SHP-2 sgRNA/Cas9 CD19 CAR	↑ degranulation & IL-2 production	Ruella et al., 2020
	CD4-Cre; SHP-2^fl/fl^	↓ colitis-associated colorectal cancers	Liu et al., 2017
		↑ Th1 differentiation & IFNγ production	Liu et al., 2017
		↑ response to colon cancers	Zhao et al., 2019

### Regulating T Cell Activation Thresholds to Improve Adoptive Cell Transfer

Limitations of ACT using TILs or engineered T cells are largely imposed by resistance mechanisms of cancer cells and their evasion from immune surveillance. Tumor antigens can be divided into tumor-specific antigens (TSAs) and tumor-associated antigens (TAAs) (Magalhaes et al., 2019; Janelle et al., 2020). TSAs refer to antigens and neoantigens that often newly arise from acquired genetic variants. As these are antigens that the adaptive immune system has not experienced previously, TSAs usually elicit vigorous immune responses that are specific to the cancer cells (Robbins et al., 2013; Lu et al., 2014) and are thought to elicit fewer on-target off-tumor effects in the patient because their expression is restricted to the tumor (Wang and Cao, 2020). TAAs on the other hand include embryonic/differentiation antigens and overexpressed self-antigens. TAAs are widely used as targets for immunotherapy because they often expressed across several cancer types (Janelle et al., 2020). However, it is presumed that overexpressed TAAs induce weaker T cell responses because T cells expressing TCRs with strong affinity to self-antigens would be eliminated by negative selection during development in the thymus (Aleksic et al., 2012). Since TAAs are frequently used as target antigens for ACT, enhancing the responses of T cells that express low-affinity TCRs would potentially improve immunotherapy of cancer (Figure 5). Targeting the T cell activation threshold in a cell intrinsic manner could expand the TCR repertoire available to ACT. PTPs that limit the threshold of TCR activation in response to low-affinity antigens are interesting potential targets in this regard.

PTPN22 is important for regulating TCR sensitivity to low-affinity agonists, and murine PTPN22-deficient OT-1 CD8 T cells were more permissive for the production of an effector response to a self-antigen (Salmond et al., 2014). Moreover, weak agonists stimulated substantially more *PTPN22*^–/–^ OT-1 CD8 T cells to produce IFNγ, TNFα, and GM-CSF (Salmond et al., 2014). Similarly, knockout of PTPN2 in OT-1 CD8 T cells resulted in enhanced cell proliferation and this effect was more pronounced in cells that were stimulated with lower-affinity peptides (Wiede et al., 2011). Studies have shown that deletion of PTPN22 or PTPN2 in CD8 T cells improved tumor clearance in a number of mouse tumor models (Brownlie et al., 2017, 2019; LaFleur et al., 2019; Wiede et al., 2020). In particular, responses to weak affinity tumor antigens were enhanced by knockout of PTPN22 which suggests that this strategy might be beneficial in promoting T cell responses to weaker TAAs (Brownlie et al., 2017, 2019).

Several studies have assessed the influence of SHP-1 on modulating the ability of T cells expressing TCRs of different affinities to control tumor cells. Hebeisen et al. found that the effectiveness of TCR-engineered CD8 T cells to kill tumors was limited by two different mechanisms (Hebeisen et al., 2013). The first was characterized by preferential expression of the PD-1 inhibitory receptor within T cells expressing the highest supraphysiological affinity TCR, and T cells with this variant TCR benefited most from PD-L1 blockade. The second was associated with the progressive increase of SHP-1 expression in a TCR affinity-dependent manner. In contrast to PD-L1 blockade, inhibition of SHP-1 (and partially SHP-2) using the PTP inhibitor sodium stibogluconate (SSG) resulted in increased degranulation and cytotoxicity of engineered T cells for all TCR affinity variants. These results suggest that SHP-1 may play a dual role and restrict not only T cell signaling of lower affinity TCRs (Stefanova et al., 2003), but also of higher and supraphysiological affinities. This role seems to be independent of PD-1 signaling because only T cells with the highest affinity TCR variant benefited from PD-L1 blockade. Together these results indicated that targeting SHP-1 in T cells with engineered TCRs can augment their functional efficacy. However, another study demonstrated that although SHP-1 knockdown functionally enhanced low-affinity T cells, it showed limited therapeutic benefit for the treatment of B16 melanoma cells *in vivo* (Snook et al., 2020). A partial CRISPR-mediated knockout of SHP-1 in the SUP-T1 cell line resulted in increased phosphorylation of CD3 chains and of ERK1/2 in all NY-ESO1-TCR affinity variants used, with the exception of the lowest affinity variant (Presotto et al., 2017). TCR variants considered to have an optimal affinity for pMHC showed the greatest increase in pERK1/2 in SHP-1 knockout cells.

There appears to be an optimal window for TCR affinities and increasing TCR-pMHC affinities and binding half-lives above a natural level can lead to less functional T cells (Kalergis et al., 2001; McMahan et al., 2006; Thomas et al., 2011). It seems that maximal biological activity occurs between a well-defined affinity window with K_D_ ranging from 5 to 1μM (Schmid et al., 2010; Irving et al., 2012), but this could differ between various TCRs. SHP-1 seems to restrict not only signaling of lower-affinity TCRs, but also of high-affinity TCRs (Hebeisen et al., 2013). Thus, deletion of SHP-1 in human T cells would need to be tested for each TCR and whether functional enhancement occurred would need to be monitored. Furthermore, SHP-1 deficient CD4 T cells produced more IL-2 and in these cells loss of SHP-1 obviated the requirement for CD28 co-stimulation (Sathish et al., 2001b). Engagement of co-stimulatory receptors such as CD28, LFA-1 and CD2 can significantly lower the threshold of responsiveness of the TCR (Viola and Lanzavecchia, 1996; Bachmann et al., 1997, 1999). Targeting SHP-1 in T cells using shRNAs or CRISPR might be a promising strategy to improve adoptive T cell transfer. Indeed, deletion of SHP-1 using shRNAs T cells demonstrated enhanced cytotoxicity *in vitro* (Stromnes et al., 2012).

Although several studies have shown that the phosphatases SHP-1 and SHP-2 have overlapping substrate specificities, other studies have indicated that they preferentially co-localize with the TCR and PD-1, respectively (Yokosuka et al., 2012; Presotto et al., 2017). Upon PD-1-ligand interaction, PD-1 and the TCR form microclusters which downregulate TCR downstream signaling by recruiting SHP-2 (Yokosuka et al., 2012). PD-1/PD-L1 and TCR complexes co-localize at the membrane and together exclude CD45 (Carbone et al., 2017). This might shift the balance in favor of PD-1 signaling and attenuate TCR signaling. It was suggested that PD-1 might increase the threshold that needs to be overcome by TCR stimulation to initiate signals (Celis-Gutierrez et al., 2019). Thus, SHP-2 deficiency might also result in enhancement of TCR activity by lowering the activation threshold. However, CRISPR-mediated knockout of SHP-2 in SUP-T1 cells did rather decrease ERK1/2 phosphorylation using TCRs with increasing affinities and had no impact on proximal TCR/CD3 signal initiation (Presotto et al., 2017). In summary, although knockout of some PTPs to lower the threshold for T cell activation by tumor antigens might be a viable way to improve T cell-mediated responses to cancer cells, there remain questions about whether such a strategy would be suitable for TCRs of all affinities or whether it would benefit only a subset of tumor specific T cells.

### Targeting Phosphatases to Mitigate T Cell Exhaustion

T cell-mediated tumor responses are complex and very high-affinity CD8 T cell responses to tumor cells can lead to tolerization in the tumor microenvironment (Janicki et al., 2008; Zahm et al., 2017). Indeed, continual or prolonged exposure to the tumor antigen can induce functional exhaustion of the T cells (Schietinger et al., 2016) and tumor-infiltrating TCR-engineered T cells can progressively lose the ability to produce IFNγ and TNFα (Stromnes et al., 2015). Exhausted T cell responses have been reported in tumor settings as well as during chronic viral infections. Upregulation of inhibitory molecules (PD-1, CTLA-4, Tim-3, LAG-3, etc.) or functional dysregulation such as decreased cytotoxicity and reduced polyfunctional cytokine expression are characteristics of exhausted T cells. Polyfunctionality describes the ability of T cells to express two or more cytokines simultaneously and polyfunctional T cells are thought to be more efficient in fighting cancer cells (Ma et al., 2013). Thus, if PTPs could be targeted in order to make T cell less prone to exhaustion and more polyfunctional, it may improve T cell function in the tumor after ACT (Figure 5).

The influence of PTPN22 in T cell exhaustion has been studied most extensively in the context of chronic viral infections. Infection of PTPN22^–/–^ and control mice with lymphocytic choriomeningitis virus (LCMV) clone 13 resulted in chronic infection of the host and PTPN22^–/–^ mice controlled the viral infection more efficiently than control mice (Jofra et al., 2017). In this context, the presence of PTPN22 was able to promote CD8 T cell exhaustion; however, this was a consequence of T cell-extrinsic effects, namely, loss of PTPN22 from other hematopoietic cells. Another study found that in PTPN22^–/–^ mice after chronic LCMV infection, PTPN22^–/–^ CD8 T cells were less exhausted and more polyfunctional (Maine et al., 2016). This was dependent on CD4 T cell help because depletion of CD4 T cells in PTPN22^–/–^ mice led to exhaustion of CD8 T cells. Interestingly, the increased prolration and inflammatory cytokine expression of PTPN22^–/–^ CD4 T cells were also regulated by T cell-extrinsic effects. Other recent work has also suggested that loss of PTPN22 might be beneficial for anti-tumor responses, since PTPN22^–/–^ mice showed increased tumor rejections in combination with anti-PD-L1 immunotherapy (Cubas et al., 2020). This effect was dependent on T cells and IFNα signaling, although the precise mechanism remains to be determined. In the same study, the frequency of the PTPN22 R620W SNP was determined in a cohort of patients with non-melanoma skin cancer and compared with healthy controls. Interestingly, the frequency of R620W was reduced in patients, suggesting a protective effect of this SNP, and homozygous patients showed improved overall survival after anti-PD-L1 therapy. The effect of the R620W SNP, however, might be cancer origin related, as another study showed that the frequency of R620W carriers was significantly increased in chronic lymphocytic leukemia (CLL) cases compared to healthy controls (Hebbring et al., 2013). Additionally, PTPN22 was overexpressed in CLL patients and PTPN22 overexpression inhibited antigen-induced apoptosis of CLL cells (Negro et al., 2012). Thus, the outcome of checkpoint inhibition therapy may depend on the PTPN22 allele expressed and the particular cancer under study.

In a comparable study, adoptive transfer of PTPN2^–/–^ or wildtype CD8 T cells into mice chronically infected with LCMV showed that the PTPN2^–/–^ CD8 T cells proliferated more and expressed higher percentages of granzyme B^+^ cells (LaFleur et al., 2019). Interestingly, TIM-3^+^ cell frequencies were also enhanced in the PTPN2^–/–^ CD8 T cell population. Killing assays using TIM-3^+^ PTPN2^–/–^ or control CD8 T cells isolated from LCMV infected mice, showed increased killing of target cells by the TIM-3^+^ PTPN2^–/–^ T cells. Consistent with this, PTPN2 deficient OT-1 CD8 T cells were superior in controlling the growth of B16-OVA melanoma cells and these T cells expressed higher frequencies of granzyme B^+^ cells. Similar results were obtained for MC38 colon adenocarcinoma cells. Another study found that PTPN2 deficient T cells were more efficient in restraining AT3-OVA mammary carcinoma cells, but the PTPN2^–/–^ OT-1 CD8 T cells expressed lower frequencies of PD-1^+^ or LAG-3^+^ cells (Wiede et al., 2020). Strikingly, PTPN2 deletion also enhanced cytotoxicity and cytokine production of Her2-specific CAR T cells. These PTPN2^–/–^ CAR T cells expressed more PD-1 and LAG-3 than control T cells, so would potentially be more susceptible to checkpoint inhibition. Thus, the question remains whether human PTPN2-deficient T cells expressing a tumor-specific CAR or TCR would similarly express increased exhaustion markers. It is possible that the higher affinity of the CAR compared to the TCR favors the generation of exhausted T cells, but this remains to be proven. Nevertheless, PTPN2 could be an interesting target for improving T cells for ACT.

CRISPR-mediated knockout of SHP-1 and SHP-2 has also been studied in CD19-specific human CAR (CAR19) T cells and resulted in higher degranulation and higher expression of IL-2 (Ruella et al., 2020). SHP-1 deficient CAR19 T cells also secreted more IFNγ, TNFα, and IL-2 and these T cells were more efficient in killing tumor cells *in vitro* and *in vivo*. From these results the authors concluded that SHP-1 and SHP-2 deficiency reduced T cell exhaustion.

In summary, targeting of several PTPs in T cells has been shown to improve T cell polyfunctionality and decrease the exhaustion of those T cells upon chronic antigen encounter. Such studies indicate that this approach might be a promising strategy either alone or in combination with checkpoint inhibitors, to improve T cell efficacy in protecting against cancers.

### Overcoming the Suppressive Tumor Microenvironment by Targeting Phosphatases

A major challenge in therapy of solid tumors is the suppressive microenvironment that can dampen T cell responses (Wellenstein and de Visser, 2018). This microenvironment can recruit suppressive regulatory T cells (Tregs) and myeloid-derived suppressor cells or produce suppressive cytokines, such as TGFβ and IL-10, that can inhibit T cell function (Figure 5). Additionally, the metabolism of cancer cells can limit oxygen and nutrients such as glucose, and can accumulate waste products such as lactate that inhibit T cells directly (Anderson et al., 2017). Cancer cells often express ligands for inhibitory receptors on T cells. For instance, expression of the PD-1 ligand, PD-L1, in several different solid tumors was associated with worse survival of cancer patients (Wang X. et al., 2016). The hostile tumor microenvironment can lead to dysfunction or exhaustion of T cells in the tumor, or can prohibit T cell infiltration into the tumor. PTPs that are involved in the regulation of sensitivity to suppressive factors in the microenvironment would be attractive targets to improve ACT. Remarkably, OT-1 T cells that lack PTPN22 were found to be more resistant to both TGFβ-mediated suppression and suppression by PTPN22-sufficient Treg cells (Brownlie et al., 2017). It was suggested that increased IL-2 production by murine PTPN22 deficient T cells helped them overcome the suppressive effects of TGFβ in the tumor microenvironment. This led to a better response of CD8 T cells to tumors and a more efficient elimination of tumor cells.

CD4 T cells can be beneficial for cancer therapy as they can enhance CD8 T cell-mediated elimination of cancer cells (Li et al., 2016). Interestingly, deletion of PTPN2 in CD4 T cells led to increased frequencies of Th1 and Th17 cells and the loss of Treg cells. When re-stimulated *in vitro*, PTPN2^–/–^ CD4 T cells expressed more IFNγ and IL-17 (Spalinger et al., 2015). Given that pro-inflammatory responses are thought to be helpful in the suppressive tumor environment, deletion of PTPN2 in CD4 T cells could enhance CD8 T cell-mediated tumor elimination and might also inhibit the development of Tregs. Further studies are needed to analyze the benefits of PTPN2 loss in different T cell subpopulations.

In light of the above findings, one might consider the use of small molecule inhibitors that target phosphatases such as PTPN22 and PTPN2 as a viable therapeutic option to improve ACT. A recent study has shown that tumor clearance was improved in PTPN22^–/–^ mice when combined with anti-PD1 therapy, in support of this strategy (Cubas et al., 2020). However, such an approach requires caution as PTPN22 is expressed in all hematopoietic cells and was found to be important for conventional dendritic cell homeostasis (Purvis et al., 2020). In mice PTPN22 deficiency led to increased frequencies of Tregs in the thymus and the periphery (Maine et al., 2012; Knipper et al., 2020). Moreover, PTPN22^–/–^ Tregs were more suppressive and secreted more IL-10 than their wildtype counterparts (Brownlie et al., 2012) which might promote a more suppressive tumor environment. PTPN2 is also expressed ubiquitously and plays multiple roles in different cells (Mosinger et al., 1992; Spalinger et al., 2018) so that the use of small inhibitors targeting PTPN2 in cancer might present the risk of causing additional side-effects. In addition, PTPN2 is 72% identical to another Tyr phosphatase, PTP1B, within the catalytic domain (Romsicki et al., 2003), which might pose problems for the development of inhibitors specific for PTPN2.

Small molecule inhibitors that target the action of the phosphatases SHP-1 and/or SHP-2 are currently undergoing extensive clinical trials for efficacy in cancer treatment (Table 4). Interestingly, the absence of SHP-1 in CD8+ T cells allowed them to resist suppression by Treg activity in a T cell-intrinsic manner, which may be crucial to survival of those cells once they enter the tumor microenvironment (Mercadante and Lorenz, 2017). However, inhibition of SHP-1 in Tregs led to increased suppressive function and TCR-antigen presenting cell (APC) conjugate formation (Iype et al., 2010). This is an important aspect when using small molecule inhibitors because they could also act on Tregs, thereby enhancing suppression in the tumor microenvironment. A preclinical study with the SHP-1 inhibitor TPI-1 showed anti-tumor effects in established B16 melanomas (Kundu et al., 2010). However, several phase I studies with the PTP inhibitor SSG in combination with IFNα showed no clinical response (Naing et al., 2011; Yi et al., 2011). Being phase I studies, anti-tumor effects were measured, but were not the primary focus. Moreover, SSG is not specific for SHP-1 but also inhibits SHP-2 (Pathak and Yi, 2001). Off-target effects might be one explanation for the lack of clinical efficacy. Another explanation could be that SHP-1 expression is altered in many malignancies, and small molecule inhibitors might influence not only SHP-1 activity in hematopoietic cells but also in the tumor cells themselves. A better approach may be to specifically target SHP-1 in CD8 T cells. Indeed, it was shown that SHP-1^–/–^ CD8 T cells proliferated better and had improved cytolytic activity *in vitro*, and ultimately showed improved clearance of leukemia cells in a preclinical adoptive T cell therapy mouse model (Stromnes et al., 2012). In this study, a higher percentage of SHP-1^–/–^ CD8 T cells secreted IFNγ and TNFα, which might be one of the mechanisms by which SHP-1^–/–^ CD8 T cells were able to eliminate tumor cells more efficiently. SHP-1-deficient CD8 T cells produced more IL-2 and formed more stable and long-lasting conjugates with APCs (Sathish et al., 2007). However, another study could not confirm better B16 melanoma elimination by SHP-1-deficient OT-1 CD8 T cells (Snook et al., 2020). These discrepancies may be a reflection of different sensitivity to elimination by ACT in the different tumor models used in these studies. However, the latter study found that combination of transfer of SHP-1^–/–^ T cells and anti-PD-1 treatment improved control of tumor growth indicating that therapies that combine inhibitory molecules blockade and ACT with T cells lacking PTPs could be a beneficial strategy for cancer treatment. This approach would need further testing in preclinical studies.

**TABLE 4:** Clinical trials of SHP-1 and SHP-2 phosphatase inhibitors.

**Trial number**	**Compound**	**Target(s)**	**Disease**	**Status**	**References**
NCT00629200	Sodium stibogluconate	SHP-1 and SHP-2	Various solid tumors	Phase I completed, no objective response, adverse events in up to 68% of patients	Naing et al., 2011
NCT00498979	Sodium stibogluconate	SHP-1 and SHP-2	Malignant melanoma	Phase I completed, no objective response, dose-limiting toxicities	Yi et al., 2011
NCT03443622	SC-43	SHP-1	Refractory solid tumors	Phase I completed	
NCT00311558	Sodium stibogluconate	SHP-1 and SHP-2		Phase I completed	Yi et al., 2011
NCT03114319	TNO155	SHP-2	Advanced solid tumors	Phase I recruiting	
NCT04000529	TNO155	SHP-2	Advanced solid tumors	Phase I recruiting	
NCT04330664	TNO155	SHP-2	KRAS G12C mutation cancers	Phase I recruiting	
NCT03565003	JAB-3068	SHP-2	Advanced solid tumors	Phase I/IIa recruiting	
NCT03518554	JAB-3068	SHP-2	Advanced solid tumors	Phase I recruiting	
NCT04045496	JAB-3312	SHP-2	Advanced solid tumors	Phase I recruiting	
NCT03634982	RMC-4630	SHP-2	Relapsed/Refractory solid tumors	Phase I recruiting	
NCT03989115	RMC-4630	SHP-2	Relapsed/Refractory solid tumors	Phase I recruiting	
NCT04252339	RLY-1971	SHP-2	Advanced or metastatic solid tumors	Phase I recruiting	

SHP-2 deficiency in CD4 T cells was found to augment colitis and reduced the incidence of colitis-associated colorectal cancers (Liu et al., 2017). SHP-2 deficiency also resulted in increased Th1 differentiation and IFNγ production. A recent study using the allosteric SHP-2 inhibitor SHP099 showed reduced tumor growth in an anti-PD-1-resistant non-small cell lung cancer mouse model (Chen et al., 2020). They found a higher percentage of CD8 T cells in tumors treated with the inhibitor. In a xenograft melanoma tumor model, tumor growth was inhibited by the SHP-2 inhibitor 11a-1 (Zhang et al., 2016). Moreover, tumor growth of colon cancer cells was reduced after treatment with different SHP-2 inhibitors (Zhao et al., 2019). CD8 tumor infiltrating lymphocytes from mice treated with the SHP-2 inhibitor produced more IFNγ and granzyme B. When colon cancer cells were injected into mice lacking SHP-2 in T cells, the resulting tumors were significantly smaller in the knockout mice. There are several ongoing clinical trials using SHP-2 inhibitors for cancer treatment (Table 4) but no efficacy data are yet available. However, SHP-2 is expressed in macrophages and SHP-2 inhibitors can negatively regulate suppressive M2-type tumor-associated macrophages (Chen et al., 2020) which could positively influence the outcomes for cancer treatment. Interestingly, two recent mouse studies confirmed an advantage in controlling tumor cell growth when using SHP-2 inhibitors in combination with anti-PD-1/PD-L1 therapy (Zhao et al., 2019; Chen et al., 2020). These results again indicate that combination therapies targeting PTPs and inhibitory molecule blockade might be an effective cancer treatment. In summary, using PTPs to improve the function of T cells in the tumor microenvironment or to make the cells less prone to tumor-intrinsic inhibitory mechanisms could be a valuable tool to improve T cell-mediated killing of tumor cells.

### Other Challenges for Improving Adoptive T Cell Therapy

*Ex vivo* manufacturing of T cells on a commercial scale remains a challenge. Problems include the variability of the starting material between patients and limited understanding of the parameters that are necessary to produce high-quality T cells in sufficient numbers for the transfer (Amini et al., 2020). There is some evidence from animal models suggesting that multiple doses of adoptively transferred T cells are superior to a single infusion, and therefore, expanding as many cells as possible would be an advantage. PTPs are involved in the regulation of T cell proliferation after TCR stimulation. For instance, the presence of murine PTPN2 attenuated T cell activation and proliferation *in vitro*, indicating that deletion of PTPN2 in T cells could lead to better proliferation and increased cell numbers which would be advantageous. Additionally, PTPN22 deficient murine T cells that were adoptively transferred into immunodeficient lymphopenic hosts showed more proliferation (Knipper et al., 2020) and patients are frequently rendered lymphopenic before ACT to improve engraftment of the transferred cells.

It is still out for debate which T cell subpopulation is more efficient in eliminating tumor cells and additionally form long-lasting memory responses after adoptive transfer into the cancer patient. Some studies show that effector T cells are most efficient in eliminating tumor cells initially, but fail to persist *in vivo* or form memory responses (Warren et al., 2010). This might be due to their terminal differentiation stage and consequently rapid exhaustion as a result of the extensive *in vitro* expansion protocol. A search for the T cell subpopulation with the highest proliferative potential has led to the identification of the stem cell-like memory T cell (T_SCM_) subpopulation (Gattinoni et al., 2009, 2011). These memory T cells have a phenotype similar to naïve T cells, but they co-express memory markers, for instance CD95 and IL-2Rβ (Gattinoni et al., 2011). T_SCM_ cells represent the least differentiated memory subpopulation and undergo extensive proliferation in response to the homeostatic cytokines IL-15 and IL-7. In a humanized mouse model T_SCM_ cells were more efficient in eliminating mesothelioma tumor cells.

PTPN22^–/–^ mice (Hasegawa et al., 2004; Brownlie et al., 2012) and mice that lack PTPN2 in T cells (Wiede et al., 2011, 2017b) show increased expansion of the effector and memory T cell compartment. Conditional knockout of SHP-1 in CD8 T cells resulted in greater expansion of the cells after stimulation with low peptide concentrations (Fowler et al., 2010). Additionally, SHP-1 deficiency limited the formation of short-lived effector cells and did not influence the generation of long-lived memory cells. In contrast, deficiency of the phosphatase SHP-2 in CD8 T cells did not affect the formation of memory T cells (Miah et al., 2017). Therefore, targeting PTPs to increase the proportion of memory T cells, and especially T_SCM_ cells, in the T cell product or after adoptive transfer could further improve ACT. In the future, more detailed analysis of the memory populations and the differentiation state of the cells is necessary to determine the potential of targeting PTPs to improve in vitro generation of T cells for adoptive T cell therapy.

## Concluding Remarks

It is clear that phosphatases are essential for regulating T cell responsiveness. However, with the exception of CD45, T cell restricted loss of the other PTPs discussed here tended to have rather subtle effects. Nevertheless, the experience of GWAS studies that have linked mutations in multiple PTP genes with increased susceptibility to a wide variety of autoimmune diseases, point to the importance of PTPs in maintaining immune cellular homeostasis. These genetic PTP variants may affect the behavior of multiple hematopoietic (and possibly non-hematopoietic) cell types, as most are widely expressed, which undoubtedly contributes to autoimmunity. However, they also have clear effects on T cell behavior and T cells are generally considered to be key drivers of autoimmunity. Important features of autoimmune T cell behavior are their resistance to cellular exhaustion in the face of persistent antigen, their persistence in hostile inflamed tissue sites, and their resistance to both immunosuppressive cytokines, including TGFβ, and to the action of Tregs. A wealth of experimental evidence now suggests that these undesirable features of autoimmune T cells can be reproduced in a cell intrinsic fashion by the selective removal of any one of several PTPs. On the flip side, ACT experiments with T cells lacking these PTPs have shown improved anti-tumor activity in a variety of mouse models. Taking these lessons learned from studying autoimmune T cells, therefore, has interesting potential for the improvement of human T cell adoptive cell therapy and we await confirmation that these lessons will indeed be beneficial in a clinical setting.

## Author Contributions

PC-S, ART, and SP performed the bibliographical searches and wrote the manuscript. RZ conceived the review, supervised the writing, and revised the manuscript. All authors read and approved the final version of the manuscript.

## Conflict of Interest

The authors declare that the research was conducted in the absence of any commercial or financial relationships that could be construed as a potential conflict of interest.
